# Insights into membrane association of the SMP domain of **extended synaptotagmin**

**DOI:** 10.1038/s41467-023-37202-8

**Published:** 2023-03-17

**Authors:** Yunyun Wang, Zhenni Li, Xinyu Wang, Ziyuan Zhao, Li Jiao, Ruming Liu, Keying Wang, Rui Ma, Yang Yang, Guo Chen, Yong Wang, Xin Bian

**Affiliations:** 1 State Key Laboratory of Medicinal Chemical Biology, College of Life Sciences, Frontiers Science Center for Cell Responses, Nankai University, Tianjin, China; 2 College of Life Sciences, Nankai University, Tianjin, China; 3 College of Life Sciences, Zhejiang University, Hangzhou, China; 4 College of Physical Science and Technology, Xiamen University, Xiamen, China; 5 Institute of Molecular Medicine, Renji Hospital, School of Medicine, Shanghai Jiao Tong University, Shanghai, China; 6 The Provincial International Science and Technology Cooperation Base on Engineering Biology, International Campus of Zhejiang University, Haining, China

**Keywords:** Membrane lipids, Membrane biophysics, Mechanism of action, Membranes

## Abstract

The Synaptotagmin-like Mitochondrial-lipid-binding Protein (SMP) domain is a newly identified lipid transfer module present in proteins that regulate lipid homeostasis at membrane contact sites (MCSs). However, how the SMP domain associates with the membrane to extract and unload lipids is unclear. Here, we performed in vitro DNA brick-assisted lipid transfer assays and in silico molecular dynamics simulations to investigate the molecular basis of the membrane association by the SMP domain of extended synaptotagmin (E-Syt), which tethers the tubular endoplasmic reticulum (ER) to the plasma membrane (PM). We demonstrate that the SMP domain uses its tip region to recognize the extremely curved subdomain of tubular ER and the acidic-lipid-enriched PM for highly efficient lipid transfer. Supporting these findings, disruption of these mechanisms results in a defect in autophagosome biogenesis contributed by E-Syt. Our results suggest a model that provides a coherent picture of the action of the SMP domain at MCSs.

## Abstract (web summary)

The SMP domain of E-Syts is a newly identified lipid transfer module with unclear mechanisms. Here, authors show that it uses its tip region to associate with the extremely curved or negatively charged membranes to extract and unload lipids.

## Introduction

Protein-mediated non-vesicular lipid transfer at membrane contact sites (MCSs), where the membranes of two different organelles are closely apposed (10–30 nm)^1,2^, plays an important role in regulating lipid homeostasis in eukaryotic cells^3,4^. One such lipid transfer module identified in recent years is the Synaptotagmin-like Mitochondrial-lipid-binding Protein (SMP) domain, which belongs to the tubular lipid-binding protein (TULIP) domain superfamily^5–8^. The SMP domain-containing proteins typically act as tethers at MCSs^8–19^. Examples of these proteins are three extended synaptotagmins (E-Syts) in mammals and their homologs in yeast, tricalbins^8,10,16,20,21^. E-Syts and tricalbins have been shown to be anchored to the tubular endoplasmic reticulum (ER) via their N-terminal hydrophobic hairpins and use their C2 domains to bind to PI(4,5)P_2_ in the plasma membrane (PM)^10,22–27^.

A crystallographic study of human E-Syt2 demonstrated that its SMP domain dimerizes in an anti-parallel fashion to form a 9-nm-long cylinder and each protomer consists of a groove lined with hydrophobic residues harboring glycerophospholipid molecules without selectivity for a specific head group^28^. However, whether this hydrophobic groove extends throughout the length of the entire SMP dimer is unknown. The bidirectional lipid transfer capacity of the SMP domain was confirmed by in vitro fluorescence resonance energy transfer (FRET)- and liposome-based lipid transfer assays^12,14,17,19,29–36^. Consistently, SMP domain-containing proteins have been reported to participate in controlling lipid homeostasis, including lipid signaling and membrane expansion, in response to acute stimuli^9,12–15,17,19,26,30,35,37–42^. For example, cells lacking E-Syts have delayed clearance of acutely accumulated diacylglycerol (DAG)^35^, sustained glucose-stimulated insulin secretion^41^, and impaired Ca^2+^-induced phosphatidylserine (PS) exposure in the PM^30^. Loss of E-Syt in Drosophila reduces PM PI(4,5)P_2_ resynthesis in photoreceptors^40^, and deletion of E-Syt3 protects against diet-induced obesity in mice^42^. Moreover, overexpression of E-Syt enhances the PM expansion driving axonal growth^37,38^, and yeast cells lacking tricalbins show defects in PM integrity upon heat shock^26^. However, compared to other well-characterized lipid transfer modules, such as oxysterol-binding protein (OSBP)-related ligand-binding domain (ORD)^43,44^, the mechanisms underlying the actions of the SMP domain are poorly understood.

We previously used DNA nanotechnology (DNA origami) to generate a DNA nanostructure comprising two DNA-ring-templated liposomes connected by a tunable DNA rod that controls the distance between the two liposomes in lipid transfer assays^31^. On the basis of this system, we suggested that the SMP domain delivers lipids as a shuttle over the typical ER-PM distance, which is approximately 15 nm on average in E-Syt1-overexpressing cells under high cytosolic Ca^2+^ levels^45^. Although the structures of the SMP domains have been determined^14,28,32,33,46^, it remains mysterious how it associates with the ER membrane and the PM to extract and unload lipids (e.g., whether the SMP domain is parallel or perpendicular to the membrane, corresponding to the lying-down or standing-up conformation). In addition, it is unclear how the 9-nm-long SMP dimer transfers lipids at occasionally observed tight ER-PM contact sites (<10 nm in distance) in cells overexpressing E-Syts or tricalbins^26,27,45^.

The SMP domain has been reported to be dispensable for the tethering function of E-Syts and its membrane association is too weak to be detected by protein-membrane interaction methods, including liposome sedimentation, liposome turbidity, and optical tweezer^10,20,23,24,30,31,35^. Here, we performed DNA brick-aided^47^ lipid transfer assays and molecular dynamics (MD) simulations to elucidate the molecular basis of membrane recognition by the SMP domain of E-Syt. Our data provide evidence that the SMP dimer uses its tip region to recognize the extremely curved subdomain of tubular ER and the acidic-lipid-enriched PM for highly efficient lipid transfer, leading to a comprehensive action model for SMP domain that takes into account its membrane association. The proposed mechanism explains the roles of E-Syts in the regulation of lipid homeostasis.

## Results

### Membrane curvature-sensing information on the SMP domain of E-Syt revealed by DNA brick-aided lipid transfer assays

**Fig. 1:**
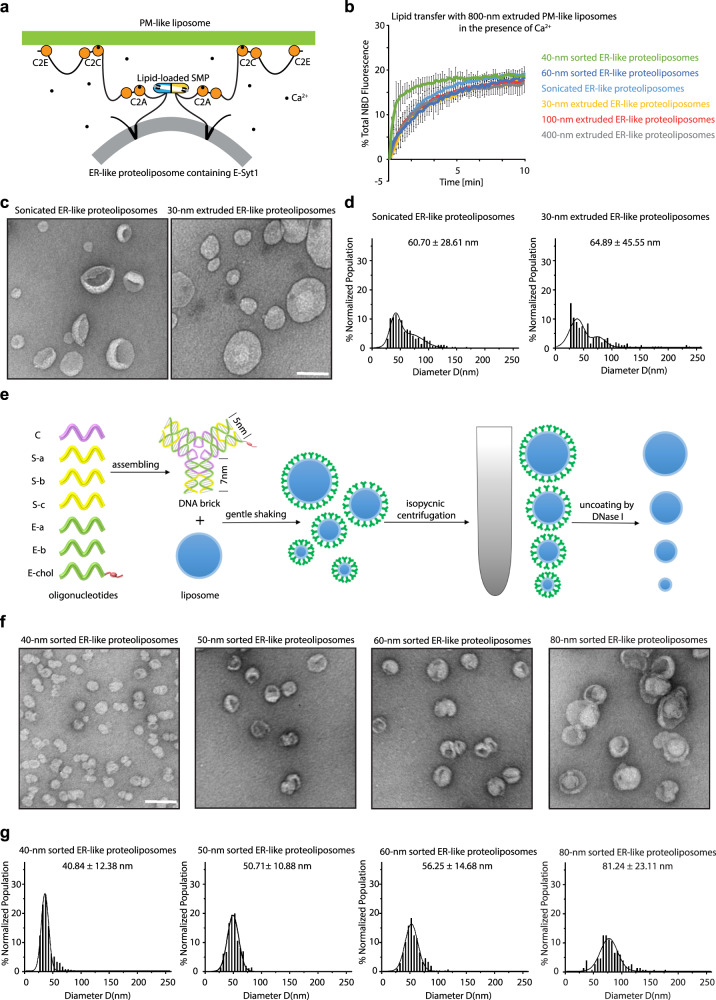
Extreme membrane curvature facilitates full-length E-Syt1-dependent lipid transfer. **a**, Schematic representation of full-length E-Syt1-dependent lipid transfer and liposome tethering in the presence of Ca^2+^. **b**, Time courses of lipid transfer between distinctly sized ER-like donor proteoliposomes containing E-Syt1 and PM-like acceptor liposomes in the presence of Ca^2+^ at room temperature as assessed by dequenching of NBD-PE fluorescence (mean ± SD, *n* = 3 independent experiments). **c**, Negative-staining TEM images showing E-Syt1-containing ER-like donor proteoliposomes prepared from sonication and extrusion through filters with 30 nm pores. Scale bar, 100 nm. **d**, Size distribution of proteoliposomes measured from negative-staining TEM images in (c). Average diameters are presented as mean ± SD (*n* = 286 and 203 proteoliposomes from left to right). **e**, Schematic of assembled DNA bricks and steps for DNA brick-assisted liposome sorting. **f**, Negative-staining TEM images showing distinctly sized E-Syt1-containing ER-like donor proteoliposomes prepared from extrusion through filters with 30 nm pores followed by DNA-brick-assisted sorting. Scale bar, 100 nm. **g**, Size distribution of proteoliposomes measured from negative-staining TEM images in F. The histograms are fitted by Gaussian functions. Average diameters are presented as mean ± SD (*n* = 152, 306, 361 and 1165 proteoliposomes from left to right). Source data are provided as a Source Data file.

The N-terminal hydrophobic hairpin of E-Syt has been reported to anchor the protein to the tubular ER^10,25–27^, but it was absent in previous FRET- and liposome-based lipid transfer assays^30,31,34–36^. To better mimic the conditions in living cells, we first purified the full-length human E-Syt1 (a.a. 1-1104) including the hydrophobic hairpin and reconstituted it into ER-like liposomes at a protein to lipid ratio of 1:500 (Fig. 1a and Supplementary Fig. 1a and b). The ER-like donor proteoliposomes containing E-Syt1, phosphatidylcholine (PC), phosphatidylethanolamine (PE), and a FRET pair (NBD-PE and Rhodamine-PE) were mixed with the PM-like acceptor liposomes composed of PC, phosphatidylserine (PS) and PI(4,5)P_2_ (Fig. 1a). In the absence of Ca^2+^, NBD-PE was efficiently quenched by Rhodamine-PE in the ER-like proteoliposomes and a modest dequenching of NBD-PE was observed (Supplementary Fig. 1c). Consistent with previous reports using the cytosolic region of E-Syt1 (E-Syt1_cyto_)^30,31,35,36,48^, in which the N-terminal region of E-Syt1 including the hydrophobic hairpin was replaced by a His tag for binding DGS-NTA(Ni) lipid in ER-like donor liposomes, the addition of Ca^2+^ strongly increased NBD fluorescence due to dilution of NBD-PE and Rhodamine-PE, which reflects their transfer from the outer leaflet of ER-like proteoliposomes to the outer leaflet of PM-like liposomes (Supplementary Fig. 1c). Basal membrane tethering by E-Syt1 revealed by the optical density at 405 nm was also enhanced by Ca^2+^ binding to the protein (Supplementary Fig. 1d). Upon adding proteinase K to the mixture, the lipid transfer and membrane tethering were completely abolished suggesting a direct role of full-length E-Syt1 in these processes in vitro (Supplementary Fig. 1).

The localization of E-Syt to the tubular ER, which varies in diameter (25–90 nm) in non-neuronal cells^49^, raises the possibility that the SMP domain is a curvature-sensing module. To test this hypothesis, we produced ER-like donor liposomes by extrusion through filters with 30 nm, 100 nm, or 400 nm pores or by sonication before reconstituting E-Syt1 into them (named 30-nm, 100-nm, or 400-nm extruded ER-like proteoliposomes or sonicated ER-like proteoliposomes, respectively). The PM-like acceptor liposomes were extruded through filters with a pore size of 800 nm to mimic the PM with low curvature. Surprisingly, detection of lipid transfer between PM-like liposomes and distinctly sized ER-like proteoliposomes did not reveal specific differences (Fig. 1a and b). However, negative-staining transmission electron microscopy (TEM) showed that the smallest ER-like proteoliposomes we used, which were prepared from sonicated or 30-nm extruded liposomes, still had heterogeneous populations with mean diameters >60 nm (60.70 ± 28.61 nm for sonicated proteoliposomes and 64.89 ± 45.55 nm for 30-nm extruded proteoliposomes, Fig. 1c, d). Therefore, it was difficult to collect curvature-sensing information on the SMP domain across the physiologically related diameter range of 25–90 nm using common lipid transfer assays.

To overcome this problem, we capitalized on a recently reported liposome-sorting strategy^47^ to obtain homogeneous sub-60-nm liposomes and applied this method to lipid transfer assays. We first constructed a three-point-star DNA nanostructure (DNA brick) consist of seven oligonucleotides (Fig. 1e and Supplementary Table 1), including a core strand (C, colored in purple, Fig. 1e), three sleeve strands (S, colored in yellow, Fig. 1e) and three edge strands (E, colored in green, Fig. 1e). One of the E strand was modified with cholesterol moiety to serve as a membrane anchor (E-chol, Fig. 1e). The DNA bricks (Fig. 1e) were assembled by thermal annealing (Supplementary Fig. 2a) and further purified by rate-zonal centrifugation (Supplementary Fig. 2b). The SDS-agarose gel analysis showed that, after coating the liposomes, the DNA bricks can be efficiently digested by DNase I treatment (Fig. 1e and Supplementary Fig. 2c).

In preparation for the lipid transfer assays with DNA brick-sorted proteoliposomes, the DNA bricks were incubated with 30-nm extruded ER-like donor proteoliposomes containing E-Syt1 at a brick to lipid ratio of 1:375 (Fig. 1e). As the spherical vesicles of different sizes had similar buoyant densities but differed in their surface-area-to-volume ratios, the coating of DNA bricks, which were highly dense, gave more density to the smaller vesicles. Accordingly, we separated the DNA brick-coated ER-like proteoliposomes by isopycnic centrifugation (Fig. 1e), and the gradient fractions (F1 to F24 from top to bottom, Supplementary Fig. 2d and e) were collected, concentrated and digested by DNase I (Fig. 1e). In agreement with a previous report using protein-free liposomes^47^, negative-staining TEM confirmed that each fraction contained uniformly sized ER-like proteoliposomes (Fig. 1f and g), which were 81.24 ± 23.11 nm diameter in F14 (named 80-nm sorted ER-like proteoliposomes), 56.25 ± 14.68 nm diameter in F16 (named 60-nm sorted ER-like proteoliposomes), 50.71 ± 10.88 nm diameter in F18 (named 50-nm sorted ER-like proteoliposomes) and 40.84 ± 12.38 nm diameter in F20 (named 40-nm sorted ER-like proteoliposomes).

To address the potential role of membrane curvature in E-Syt1 SMP-mediated lipid transfer, we mixed the sorted ER-like donor proteoliposomes containing E-Syt1 with the 800-nm extruded PM-like acceptor liposomes (Fig. 1a). In the presence of Ca^2+^, the addition of 60-nm sorted ER-like proteoliposomes resulted in an increase in NBD-PE fluorescence with a similar efficiency as the extruded or sonicated ER-like proteoliposomes, whereas the 40-nm sorted ER-like proteoliposomes dramatically accelerated the lipid transfer by E-Syt1 (Fig. 1b). These data validate the application of the DNA brick-assisted sorting technique to separate vesicles reconstituted with high-molecular-weight proteins and the use of the DNA brick-aided lipid transfer assays developed in this study for uncovering information on membrane curvature-sensing by the SMP domain of E-Syt1.

**Fig. 2:**
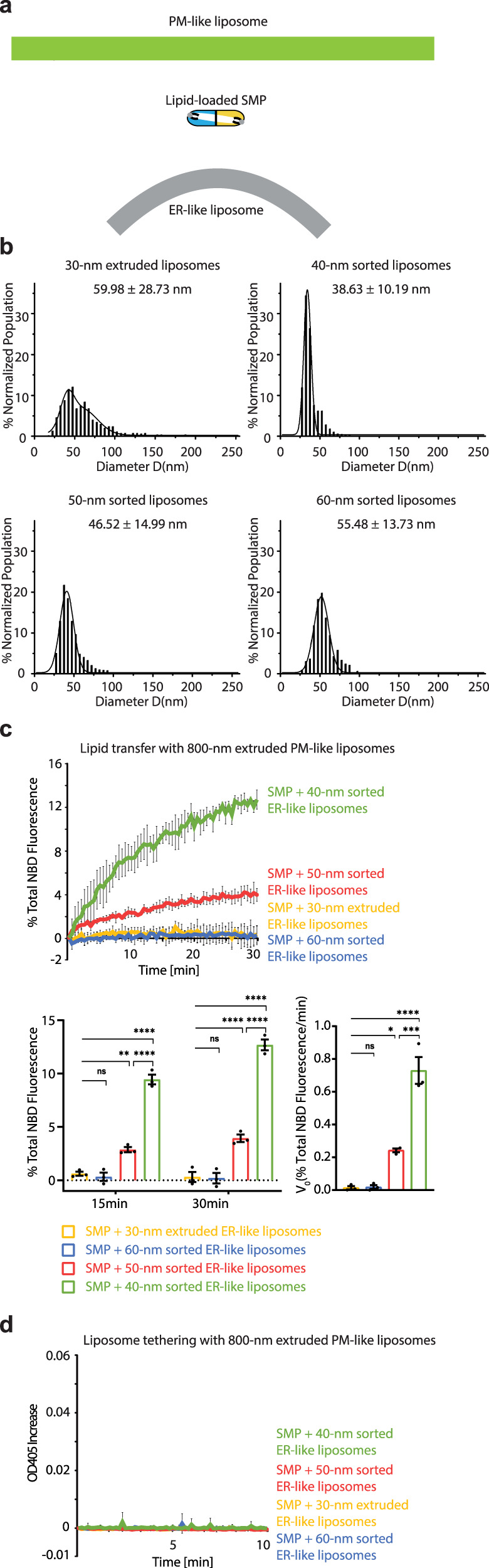
Extreme membrane curvature is essential for SMP-mediated lipid transfer. **a**, Schematic representation of SMP-mediated lipid transfer between curved ER-like and flat PM-like liposomes. **b**, Size distributions of distinctly sized ER-like donor liposomes prepared from extrusion through filters with 30 nm pores followed by DNA-brick-assisted sorting or not. Diameters of liposomes were measured from negative-staining TEM images. Average diameters are presented as mean ± SD (*n* = 548, 1959, 916, and 329 liposomes from left to right). **c**, Top, time courses of lipid transfer between distinctly sized ER-like donor liposomes and PM-like acceptor liposomes in the presence of SMP at 37 °C as assessed by dequenching of NBD-PE fluorescence. Bottom left, quantifications of NBD fluorescence after incubation for 15 min or 30 min. Bottom right, initial transfer rates. Data are presented as mean ± SD (*n* = 3 independent experiments). ns, not significant; * P < 0.05, **P < 0.01, ****P < 0.0001 by two-way ANOVA with Bonferroni’s multiple comparisons test (bottom left) or by one-way ANOVA with Bonferroni’s multiple comparisons test (bottom right). P values: 6.4 × 10 ^−3^ (bottom left, 15 min), 5.3 × 10^−5^ (bottom left, 30 min), and 0.030 (bottom right) for [(SMP + 30-nm extruded ER-like liposomes) vs (SMP + 50-nm sorted ER-like liposomes)]; 2.6 × 10^−10^ (bottom left, 15 min), 1.5 × 10^−12^ (bottom left, 30 min), and 1.3 × 10^−5^ (bottom right) for [(SMP + 30-nm extruded ER-like liposomes) vs (SMP + 40-nm sorted ER-like liposomes)]; 2.0 × 10^−8^ (bottom left, 15 min), 3.0 × 10^−10^ (bottom left, 30 min), and 2.3 × 10^−4^ (bottom right) for [(SMP + 50-nm sorted ER-like liposomes) vs (SMP + 40-nm sorted ER-like liposomes)]. **d**, Time courses of the tethering of distinctly sized ER-like donor liposomes and PM-like acceptor liposomes in the presence of SMP at 37 °C as assessed by an increase in turbidity (OD at 405 nm). Data are presented as mean ± SD (*n* = 3 independent experiments). Source data are provided as a Source Data file.

We next assessed whether the membrane curvature plays a direct role in SMP-mediated lipid transfer (Fig. 2a). The ER-like donor liposomes (PC, PE, NBD-PE and Rhodamine-PE) were obtained by extrusion through filters with a pore size of 30 nm. Similar to the 30-nm extruded E-Syt1-containing proteoliposomes, these 30-nm extruded protein-free liposomes also had a broad size distribution with a mean diameter of 59.98 ± 28.73 nm (Fig. 2b). Subsequently, we performed DNA brick-assisted sorting on 30-nm extruded ER-like liposomes. Sorted liposomes with three different diameters (55.48 ± 13.73, 46.52 ± 14.99, and 38.63 ± 10.19 nm) were collected and digested by DNase I. These liposomes were homogeneous and checked by negative-staining TEM (named 60-nm sorted, 50-nm sorted, and 40-nm sorted ER-like liposomes, Fig. 2b). After mixing the extruded or sorted ER-like liposomes with 800-nm extruded PM-like acceptor liposomes and the purified SMP domain of E-Syt1 (SMP, a.a. 134-327, Supplementary Fig. 3), we monitored the NBD-PE fluorescence and optical density at 405 nm (Fig. 2b). Consistent with previously reported results^30,31^, no lipid transfer by the SMP domain alone was observed when extruded liposomes were not tethered (Fig. 2c and d). Interestingly, the NBD-PE dequenching due to its transfer by the SMP domain slightly increased with 50-nm sorted, but not 60-nm sorted, ER-like liposomes, and 40-nm sorted ER-like liposomes produced a robust lipid transfer level (Fig. 2c). The rapid NBD dequenching signal at the beginning of the experiment was caused by the initial uptake of NBD-PE and/or Rhodamine-PE into the SMP domain. Furthermore, Ca^2+^ was not needed for SMP-dependent lipid transfer (Supplementary Fig. 4), and incorporating NBD-PE and Rhodamine-PE in either the ER-like or the PM-like liposomes resulted in a similar NBD-PE dequenching efficiency, proving that the lipid transfer is bidirectional (Supplementary Fig. 4). Notably, the SMP domain alone did not tether these liposomes (Fig. 2d) even though the protein was anchored to the 40-nm sorted DGS-NTA(Ni)-containing ER-like liposomes via its N-terminal His tag before incubating with PM-like liposomes (Supplementary Fig. 5a). In agreement with these data, the SMP domain did not sediment with 40-nm sorted ER-like liposomes or PM-like liposomes with increasing amounts of PS and PI(4,5)P_2_ (Supplementary Fig. 5b).

Taken together, these results illustrate that the membrane with extreme curvature (<50 nm in diameter) facilitates the SMP-mediated lipid transfer, and confirm the shuttle mechanism of the SMP domain over long membrane distance (> 10 nm), as SMP alone can transfer lipids between untethered liposomes.

### Charge-dependent lipid transfer by the SMP domain of E-Syt

**Fig. 3:**
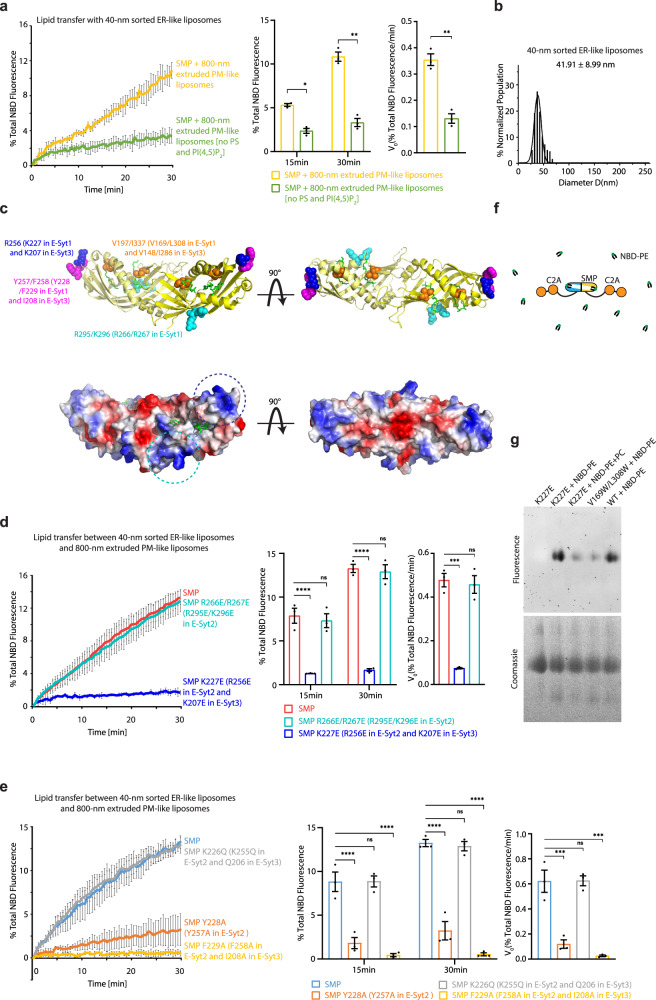
Acidic lipids in the membrane facilitate SMP-mediated lipid transfer and SMP uses its tip region to associate with the membrane. **a**, Left, time courses of lipid transfer between ER-like donor liposomes and PM-like acceptor liposomes with or without PI(4,5)P_2_ and PS in the presence of SMP at 37 °C as assessed by dequenching of NBD-PE fluorescence. Middle, quantifications of NBD fluorescence after incubation for 15 min or 30 min. Right, initial transfer rates. Data are presented as mean ± SD (*n* = 3 independent experiments). * P < 0.05; ** P < 0.01 by two-way ANOVA with Sidak’s multiple comparisons test (middle) or by two-tailed Student’s t-tests (right). P values: 0.032 (middle, 15 min), 5.0 × 10^−3^ (middle, 30 min), and 1.5 × 10^−3^ (right) for [(SMP + 80-nm extruded PM-like liposomes) vs (SMP + 80-nm extruded PM-like liposomes no PS and PIP_2_)]. **b**, Size distribution of ER-like donor liposomes prepared from extrusion through filters with 30 nm pores followed by DNA-brick-assisted sorting. Diameters of liposomes were measured from negative-staining TEM images. Average diameters is presented as mean ± SD (*n* = 789 liposomes). **c**, Ribbon representation (top left) and surface representations illustrating the electrostatic potentials (bottom left) of the crystal structure of the SMP dimer of human E-Syt2 (PDB code 4P42) rendered in PyMOL. One SMP monomer is shown in yellow and the other in pale yellow. Lipid molecules are represented as green sticks. R256 (K227 in E-Syt1 and K207 in E-Syt3) is represented as sphere in blue. R295 and K296 (R266 and R267 in E-Syt1) are represented as cyan spheres. Y257and F258 (Y228 and F229 in E-Syt1 and I208 in E-Syt3) are represented as magenta spheres. V197 and I337 (V169 and L308 in E-Syt1 and V148 and I286 in E-Syt3) are represented as orange spheres. The basic patch at the tip region is indicated by a blue dashed circle. The basic patch at the side region is indicated by a cyan dashed circle. Right, a different view of the SMP dimer. **d** and **e**, Left, time courses of lipid transfer between ER-like donor liposomes and PM-like acceptor liposomes in the presence of WT or mutated SMP at 37 °C as assessed by dequenching of NBD-PE fluorescence. Middle, quantifications of NBD fluorescence after incubation for 15 min or 30 min. Right, initial transfer rates. Data are presented as mean ± SD (*n* = 3 independent experiments). ns, not significant; *** P < 0.001, **** P < 0.0001 by two-way ANOVA with Bonferroni’s multiple comparisons test (middle) or by one-way ANOVA with Bonferroni’s multiple comparisons test (right). P values: 1.8 × 10^−5^ (middle, 15 min), 4.2 × 10^−8^ (middle, 30 min), and 2.1 × 10^−4^ (right) for [(SMP) vs (SMP K227E)]; 1.1 × 10^−5^ (middle, 15 min), 8.8 × 10^−8^ (middle, 30 min), and 7.5 × 10^−4^ (right) for [(SMP) vs (SMP Y228A)]; 1.0 × 10^−6^ (middle, 15 min), 2.8 × 10^−9^ (middle, 30 min), and 2.2 × 10^−4^ (right) for [(SMP) vs (SMP F229A)]. **f**, Schematic representation of lipid harboring by the SMP domain. **g**, Purified WT and mutated SMP-C2AB of E-Syt1 were incubated with solubilized NBD-PE or NBD-PE and POPC, run on native-PAGE, and analyzed by fluorescence (top) and Coomassie blue staining (bottom). This experiment was repeated three times with similar results. Source data are provided as a Source Data file.

In view of the low curvature of the PM, we explored whether the acidic lipids [e.g. PS and PI(4,5)P_2_], which are enriched in the PM but not the ER, participate in associating with the SMP domain for lipid transfer. The DNA brick-aided lipid transfer assays with SMP developed here (Fig. 2a) allowed us to test this plausible scenario because this system did not require PI(4,5)P_2_-dependent liposome tethering, which was a premise for observing lipid transfer by E-Syt1 in previous studies^30,31,35,36^. The assays involved the SMP, 40-nm sorted ER-like donor liposomes, and 800-nm extruded PM-like acceptor liposomes [Fig. 2a, with or without removal of PS and PI(4,5)P_2_ from PM-like liposomes]. A much lower degree of NBD-PE and Rhodamine-PE transfer by SMP between liposomes occurred with PM-like liposomes devoid of PS and PI(4,5)P_2_ (Fig. 3a and b). These results reflect that the lipids with negatively charged headgroups also function as binding sites for the SMP domain to extract and unload lipids.

Consistent with the high similarity among the corresponding domains of all three E-Syts (Supplementary Fig. 6), the SMP domains of E-Syt2 and E-Syt3 showed similar membrane curvature- and charge-facilitated lipid transfer activities (Supplementary Fig. 7).

### Association of the SMP domain with the membrane via its tip region

To gain insights into the mechanisms underlying the membrane association of the SMP domain, we first investigated the interplay between the SMP domain and the PM. According to the crystal structure of the SMP-C2AB domains of human E-Syt2^28^, there are two small basic patches in the SMP domain that have the potential to interact with the acidic lipids in the PM (tip region patch and side region patch, Fig. 3c). We mutated the key conserved positively charged residues (Fig. 3c and Supplementary Fig. 6) in these patches to negatively charged residues to assess their importance in SMP-mediated lipid transfer. The R266E/R267E double mutant in the side region did not inhibit lipid transfer when mixing mutated SMP, 40-nm sorted ER-like donor liposomes, and 800-nm extruded PM-like acceptor liposomes (Figs. 2a, 3c and d). In contrast, the lipid transfer ability of E-Syt1 SMP was blocked when K227 in the tip region was mutated to Glu (Figs. 2a, 3c-d, and Supplementary Fig. 3). Similar lipid transfer defects were also observed for E-Syt2 SMP bearing R256E and E-Syt3 SMP bearing K207E (Supplementary Fig. 7). The K226 in E-Syt1, which is less conserved among three E-Syts (K255 in E-Syt2 and Q206 in E-Syt3, Supplementary Fig. 6), was dispensable for membrane association, as the lipid transfer by SMP K226Q was similar to wild-type (WT) SMP (Fig. 3e). For full-length E-Syt1, the 40-nm sorted ER-like proteoliposomes containing E-Syt1 K227E reduced, but did not abolish, lipid transfer (Fig. 1a and Supplementary Fig. 8).

The K227E mutant did not affect the lipid harboring ability of the SMP domain of E-Syt1, as the solubilized NBD-PE was loaded onto purified WT protein and K227E mutant at a similar level based on the fluorescence of NBD-PE in the band corresponding to SMP-C2AB of E-Syt1 in native polyacrylamide gel electrophoresis (Fig. 3c, f and g). The lipid harboring of K227E mutant was also demonstrated by the ability of PC to displace preloaded NBD-PE from the protein (Fig. 3g). As a control, mutating two hydrophobic residues to bulky hydrophobic amino acids that block the hydrophobic cavity of the SMP domain (V169W/L308W) impaired lipid harboring ((Fig. 3c and g)^35^. Collectively, these results suggest that the tip region of the SMP domain drives its association with the PM via a charge-based interaction for lipid extraction and release.

**Fig. 4:**
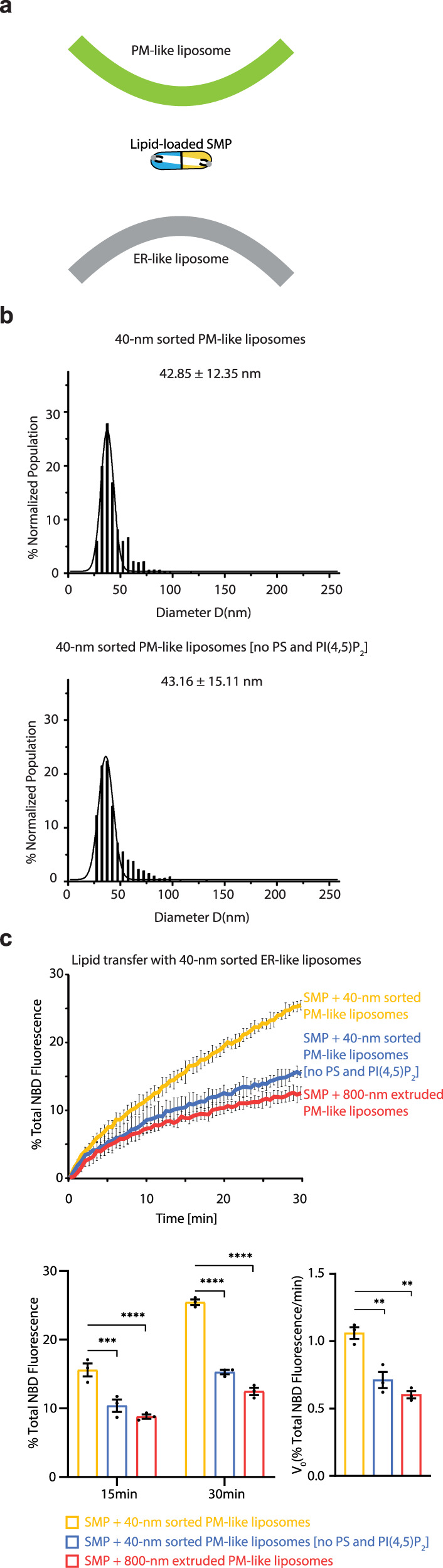
Extreme membrane curvature and acidic lipids have a synergistic effect on SMP-mediated lipid transfer. **a**, Schematic representation of SMP-mediated lipid transfer between curved ER-like and curved PM-like liposomes. **b**, Size distributions of PM-like acceptor liposomes with or without PI(4,5)P_2_ and PS prepared from extrusion through filters with 30 nm pores followed by DNA brick-assisted sorting. Diameters of liposomes were measured from negative-staining TEM images. Average diameters are presented as mean ± SD (*n* = 1258 and 1153 liposomes from left to right). **c**, Top, time courses of lipid transfer between ER-like donor liposomes and distinctly sized PM-like acceptor liposomes with or without PI(4,5)P_2_ and PS in the presence of SMP at 37 °C as assessed by dequenching of NBD-PE fluorescence. Bottom left, quantifications of NBD fluorescence after incubation for 15 min or 30 min. Bottom right, initial transfer rates. Data are presented as mean ± SD (*n* = 3 independent experiments). ** P < 0.01, *** P < 0.001, **** P < 0.0001 by two-way ANOVA with Bonferroni’s multiple comparisons test (bottom left) or by one-way ANOVA with Bonferroni’s multiple comparisons test (bottom right). P values: 2.3 × 10^−4^ (bottom left, 15 min), 2.4×10^−7^ (bottom left, 30 min), and 5.0 × 10^−3^ (bottom right) for [(SMP + 40-nm sorted PM-like liposomes) vs (SMP + 40-nm sorted PM-like liposomes no PS and PIP_2_)]; 1.8×10^−5^ (bottom left, 15 min), 1.6 × 10^−8^ (bottom left, 15 min), and 1.2 × 10^−3^ (bottom right) for [(SMP + 40-nm sorted PM-like liposomes) vs (SMP + 800-nm extruded PM-like liposomes)]. Source data are provided as a Source Data file.

For the SMP-ER association, we hypothesized that the SMP domain associates with the ER membrane in the same way as it interacts with the PM. Y257 and F258 in the tip region of E-Syt2, corresponding to Y228 and F229 in E-Syt1 and I208 in E-Syt3 (Fig. 3c and Supplementary Fig. 6), are expected to be inserted into the bilayer. The importance of the tip region for membrane association was assessed by mutating these residues to Ala. The SMP of E-Syt1 bearing Y228A or F229A (Supplementary Fig. 3) had lower lipid transfer activity (Figs. 2a, 3c and e). F258A of E-Syt2 and I208A of E-Syt3 also reduced lipid transfer by SMP (Supplementary Fig. 7). Furthermore, if the SMP domain uses the same region to recognize the extreme membrane curvature and negatively charged lipids, these two membrane features are proposed to have a synergistic effect on its lipid transfer (Fig. 4a). To test this, the PM-like acceptor liposomes consisting of PC, PS, and PI(4,5)P_2_ was extruded through filters with 30 nm pores followed by DNA brick-assisted sorting. The 40-nm sorted PM-like liposomes were collected and concentrated (42.85 ± 12.35 nm in diameter, Fig. 4b). In the lipid transfer assays, replacement of 800-nm extruded PM-like liposomes with 40-nm sorted PM-like liposomes resulted in an increase in the lipid transfer activity of SMP (Figs. 2a, 4a and c). Lack of PI(4,5)P_2_ and PS in the 40-nm sorted PM-like liposomes (43.16 ± 15.11 nm in diameter, Fig. 4b) induced a slower NBD-PE dequenching (Fig. 4a and c). These results support a model in which the SMP domain uses its tip region to associate with not only the PM but also the tubular ER for lipid transfer.

### Molecular dynamics simulations of membrane association by the SMP domain of E-Syt

**Fig. 5:**
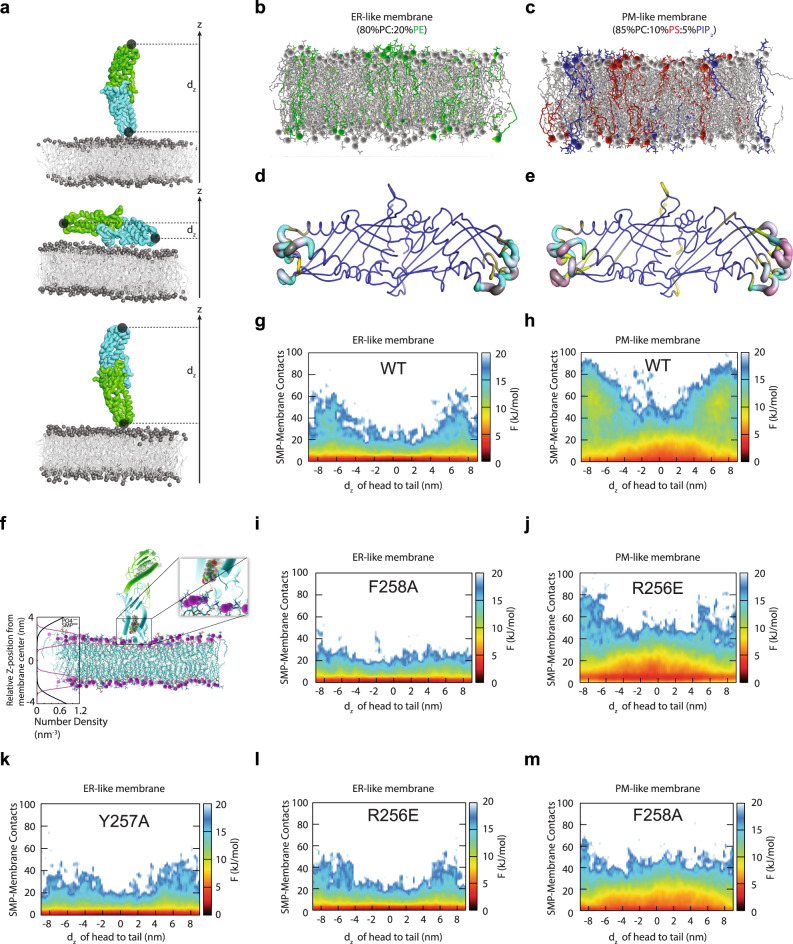
Membrane association by the SMP domain is supported by molecular dynamics simulations of E-Syt2 SMP dimer with an ER-like and a PM-like membrane. **a**, Representative conformations corresponding to the “standing-up” and “lying-down” bound states. **b**, Representative structure of an ER-like membrane model consisting of 80% POPC:20% POPE. **c**, Representative structure of an PM-like membrane model consisting of 85% POPC:10% POPS:5% PI(4,5)P_2_. **d**, **e** The membrane binding frequency of SMP residues mapped onto the crystal structure of E-Syt2 (PDB code 4P42) with the ER-like **d**, and the PM-like membrane **e**, respectively. The binding frequency is visualized by the thickness of the tube (thinner tube represents lower frequency) and different colors from blue (the lowest frequency), yellow, green, cyan, gray to magenta (the highest frequency). **f** Atomic model of SMP dimer inserting into the membrane. The depth of the insertion was obtained from the coarse-grained MD simulation. The system was energy minimized and equilibrated using all-atom MD simulation. **g**–**m** The free energy landscapes of SMP binding with the ER-like membrane **g**, SMP binding with the PM-like membrane **h**, SMP F258A mutant binding with the ER-like membrane **i**, SMP R256E mutant binding with the PM-like membrane **j**, SMP Y257A mutant binding with the ER-like membrane **k**, SMP F258A mutant binding with the PM-like membrane **l**, and SMP R256E mutant binding with the ER-like membrane **m**, as a function of the distance between the two ends of the SMP dimer projected to the membrane normal and the number of contacts between SMP and the membrane bilayer. Source data are provided as a Source Data file.

To further understand the molecular mechanism underlying the membrane association of the SMP domain, we performed molecular dynamics (MD) simulations of SMP dimer starting from the crystal structure of E-Syt2^28^ (Fig. 5a) and investigated its interactions with an ER-like bilayer consisting of PC and PE (Fig. 5b) and a PM-like bilayer consisting of PC, PS and PI(4,5)P_2_ (Fig. 5c). To reach a longer timescale, we applied the latest optimized version of the coarse-grained (CG) Martini force field, which has been widely used to study protein-lipid interactions^50,51^. This allowed us to accumulate hundreds of microsecond data with dozens of simulations (Supplementary Table 2) that reflect the membrane binding frequency of the residues of the SMP domain to the ER-like membrane model and the PM-like membrane model (Fig. 5d and e). We observed that the SMP dimer mostly used the residues located at the two tips to interact with the membrane bilayer in both the ER-like and PM-like membrane models (Fig. 5d and e). The MD simulations also showed that the tip region of SMP dimer inserted into the bilayer to place the lipid binding pocket in close proximity to its cargo lipids (Fig. 5f).

These membrane bindings were further supported by projecting the MD trajectories onto the two-dimensional free energy surfaces as a function of the distance between the two ends of the SMP dimer projected to the membrane normal (d_z_) and the number of contacts between SMP and the membrane bilayer (Fig. 5a, g and h). Given that the structure of the SMP dimer is symmetrical, the free energy surfaces were, as expected, almost symmetric along d_z_, indicating that the MD simulations converged well. The free energy surfaces revealed two free energy minima corresponding to the standing-up conformational state of SMP dimer on the membrane surface with either one of its tips interacting with the membrane surface (Fig. 5a, g and h). Collectively, these data confirmed that the SMP dimer prefers to bind perpendicularly to the membrane.

The comparison of the MD results suggested that the membrane binding of SMP was weaker in the uncurved ER-like bilayer model than that in the flat and acidic PM-like bilayer model (Fig. 5g, h). In principle, the MD simulations should allow us to simulate the SMP binding to highly curved vesicles. However, this is very computationally expensive, making it very challenging to get sufficient statistics. Alternatively, to further support our model, we performed five additional sets of CG MD simulations of the Y257A and F258A mutants of E-Syt2 SMP (corresponding to Y228A and F229A in E-Syt1 and I208A in E-Syt3, Fig. 3c and Supplementary Fig. 6) and the R256E mutant of E-Syt2 SMP (corresponding to K227E in E-Syt1 and K207E in E-Syt3, Fig. 3c and Supplementary Fig. 6) in the in the ER-like and PM-like membranes. All of these mutants, which impaired the lipid transfer activity of the SMP domain in vitro (Fig. 3 and Supplementary Fig. 7), had a significant decrease in the membrane binding probability of the SMP tip region (Fig. 5i–m).

The critical roles of the tip regions of SMP dimer in associating with the membranes lead to a plausible scenario in which the SMP dimer acts a static “tunnel” for lipid transfer at tight MCSs (<10 nm in distance). However, this hypothesis was not supported by our explicit solvent all-atom MD simulations (see Methods and Supplementary Table 2), which showed that the solvent-exposed hydrophobic channel in SMP dimer observed in the crystal structure^28^ was closed due to strong hydrophobic interactions and there was very likely a high energy barrier in the dimer interfacial region to prevent the sliding of the cargo lipid from one end to the other (Supplementary Fig. 9 and Supplementary Movie 1).

### Role of lipid transfer by the SMP domain in autophagosome biogenesis

**Fig. 6:**
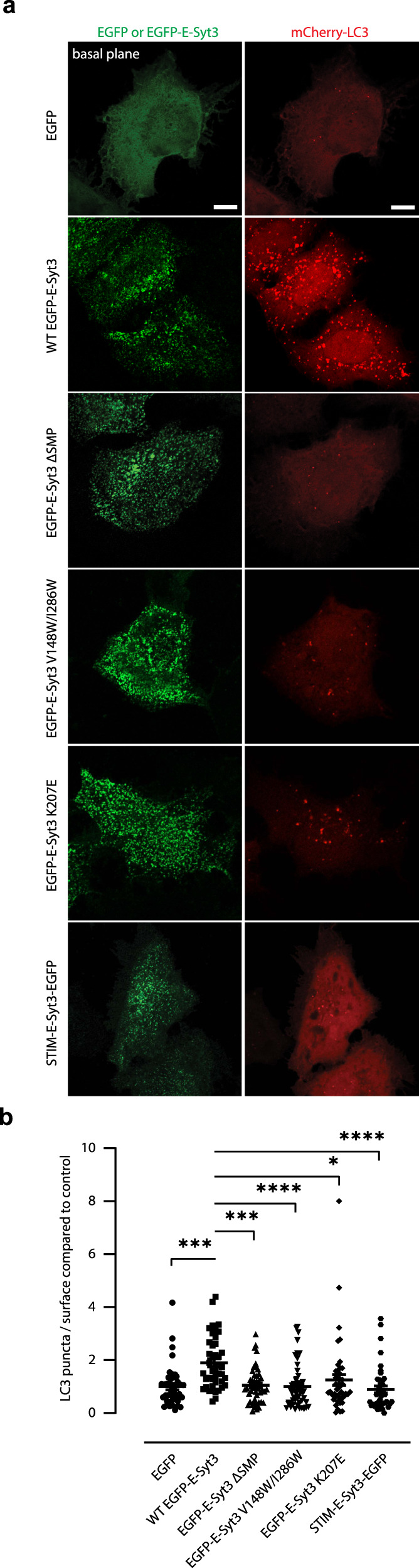
Lipid transfer by E-Syt3 contributes to autophagosome biogenesis. **a** Confocal images of HeLa cells co-expressing mCherry-LC3 and WT or mutated EGFP-E-Syt3. Scale bar, 10 μm. **b** Quantification of LC3 puncta per area compared to control. Data are presented as mean ± SEM (*n* = 42, 42, 47, 51, 45, and 42 cells from left to right). *P < 0.05, *** P < 0.001, ****P < 0.0001 by one-way ANOVA with Bonferroni’s multiple comparisons test. *P* values: 2.4 × 10^−4^ [(EGFP) vs (WT EGFP-E-Syt3)], 3^4 ^ ×  10^−4^ [(WT EGFP-E-Syt3) vs (EGFP-E-Syt3 ΔSMP)], 8.9 × 10^−5^ [(WT EGFP-E-Syt3) vs (EGFP-E-Syt3 V148W/I286W)], 0.020 [(WT EGFP-E-Syt3) vs (EGFP-E-Syt3 K207E)], 1.9 × 10^−5^ [(WT EGFP-E-Syt3) vs (STIM-E-Syt3-EGFP)]. Source data are provided as a Source Data file.

Finally, we validated the importance of SMP-membrane association in the actions of E-Syts in cells. The hydrophobic hairpins, SMP, C2A, and C2B domains are highly conserved in all three E-Syts^10,28^. Fluorescence microscopy analyses of E-Syt-expressing cells revealed that E-Syt1 translocates to ER-PM contact sites upon cytosolic Ca^2+^ elevation via regulation by its C2C domain, whereas E-Syt2 and E-Syt3 are constitutive ER-PM tethers even at resting Ca^2+^ levels^10,22,24^. A previous study^39^ showed that E-Syt2 or E-Syt3 interacts with the class 3 PI3kinase complex (PI3KC3) partner VMP1 at ER-PM contact sites to regulate autophagy-associated PI3P synthesis, which is engaged in autophagosome biogenesis. Overexpression of E-Syt2 or E-Syt3, but not E-Syt1, in HeLa cells significantly increased the number of phagophore and autophagosome marker LC3 puncta even under fed conditions (Fig. 6 and Supplementary Fig. 10a)^39^.

To better understand the role of E-Syt3 in autophagy, we overexpressed E-Syt3 construct lacking the SMP domain (E-Syt3 ΔSMP) or with mutated residues lining the lipid-harboring cavity of the SMP domain (V148W/I286W, corresponding to V169W/L308W in E-Syt1, Fig. 3c). No increase in LC3-positive structures was observed in these cells (Fig. 6). Therefore, the lipid transfer by E-Syt3 contributes to autophagosome biogenesis, which may be explained by the regulation of ER-PM contact site-associated PI3P synthesis. Importantly, when we mutated K207 in E-Syt3 to Glu (E-Syt3 K207E, corresponding to E-Syt1 K227E and E-Syt2 R256E, Fig. 3c and Supplementary Fig. 6), the overexpression of this mutant still increased and stabilized ER-PM contacts similar to WT E-Syt3 but failed to induce the formation of LC3 fluorescence puncta (Fig. 6). We also replaced the hydrophobic hairpin of E-Syt3 with a single transmembrane domain of STIM1 (STIM-E-Syt3) to further test the importance of specific ER localization of E-Syt in its physiological function. Unlike WT E-Syt3, STIM-E-Syt3 had no colocalization with another lipid transporter and ER-PM tether, ORP5^52–54^, and failed to induce the formation of LC3 fluorescence puncta, though it still increased and stabilized ER-PM contacts (Fig. 6 and Supplementary Fig. 10b). The hydrophobic hairpin of E-Syt itself is dispensable for its lipid transfer activity, as the purified His-tagged E-Syt1_cyto_ had comparable lipid transfer and membrane tethering levels as full-length E-Syt1 (Supplementary Fig. 1). These results confirm that an association of the tip region of SMP dimer with the acidic lipids in the PM and the subdomain of ER is required for its lipid transfer activity in cells.

## Discussion

A growing number of SMP domain-containing proteins have been found and reported to mediate non-vesicular lipid transfer at MCSs to control lipid homeostasis in cells^8–17,19,21,26,30,32–37,40,41^. The ER-PM tethering proteins, E-Syts (tricalbins in yeast), are better characterized members of the SMP family proteins^8,10,16,22–24,26–28,30,31,34–41,45^. However, it is technically challenging to investigate how E-Syt SMP dimer associates with membranes to extract and unload lipids because they do not stably interact with membranes^23,30,31^. In this study, we applied a recently developed DNA brick-assisted liposome sorting technique^47^ to in vitro lipid transfer assays and performed in silico MD simulations to study the mechanisms underlying the membrane association by the SMP domain of E-Syt. Unlike liposomes produced by extrusion or sonication, which have broad size distributions with mean diameters >60 nm, the liposomes coated with DNA bricks can be sorted into different homogeneous populations with mean diameters from 30 to 130 nm. Given that N-terminal hydrophobic hairpin localizes E-Syt to the tubular ER (25–90 nm in diameter)^10,25–27,49^, our DNA brick-aided lipid transfer system offers a solution for assessing the curvature-dependent activity of the protein across a physiologically relevant curvature range.

**Fig. 7:**
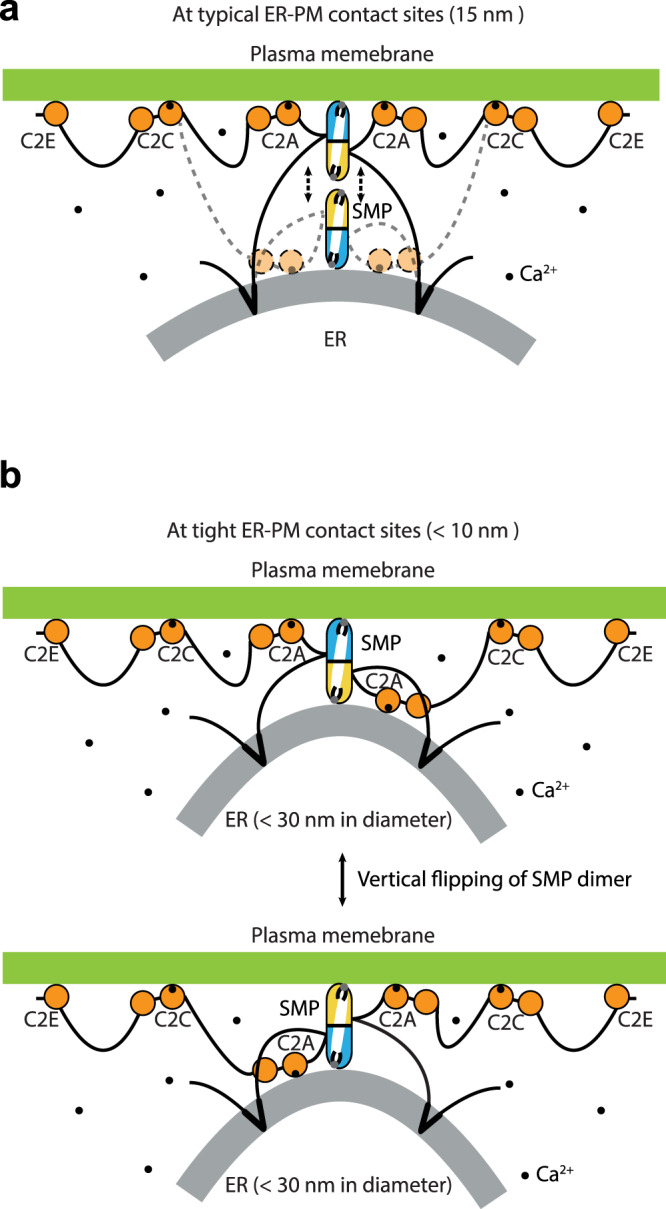
Model of SMP-mediated lipid transfer at ER-PM contact sites. E-Syt1 is anchored to the tubular ER via its N-terminal hydrophobic hairpin and interacts with the PM by its Ca^2+^-binding C2C and C2E domain. The tip region of the SMP dimer associates with the subdomain of the tubular ER and acidic-lipid-enriched region in the PM for transferring lipids as a “shuttle” at typical ER-PM contact sites **a** and as a “screw propeller” **b** at tight ER-PM contact sites. The Ca^2+^-binding C2A domain is in close proximity to the SMP domain and binds to either the ER or PM to help the SMP domain stay at the membrane. The Ca^2+^ ions are shown as small black circles.

Here, we suggest that both the extreme curvature of ER-like liposomes (<50 nm in diameter) and the negatively charged lipids in PM-like liposomes are critical for efficient lipid transfer between them by the E-Syt1 SMP domain alone. The requirement of acidic lipids for lipid transfer by the SMP domain of tricalbin 3 has also been reported in a previous study^34^. In addition, consistent with our previously proposed shuttle model (Fig. 7a)^31^, our in vitro data showed that the E-Syt1 SMP domain alone delivers lipids between ER-like and PM-like liposomes without tethering them. For full-length E-Syt1, reconstituting it into larger ER-like liposomes (>50 nm in diameter) reduced the lipid transfer activity in the presence of Ca^2+^, but the activity was not negligible. We propose that the Ca^2+^- and membrane-binding C2A domain that is in close proximity to the SMP domain helps it stay at the lowly curved membrane surface for lipid extraction and release (Fig. 7).

As all three E-Syts are similar in regard to the corresponding domains, we inspected the structure of the SMP domain of E-Syt2^28^ and found a small basic patch at its tip region. An important finding of the analyses of this region is that the SMP domain associates with the acidic lipids in the PM through the positively charged residues in this basic patch, which is not expected to be strong enough for a stable interaction (Fig. 7). For the SMP-ER association, it is plausible that the ER membrane is also recognized by the tip region of the SMP domain (Fig. 7). This was found to be the case in our experimental and in silico analyses, as the curvature-facilitated lipid transfer by the SMP domain and its membrane binding frequency to the ER-like membrane model were reduced when the large hydrophobic residues at its tip region were substituted with Ala. According to our MD simulations (Fig. 5f), we also propose that similar to the model of Mdm34^55^, the insertion of the tip region of the E-Syt SMP domain into the bilayer allows the lipid binding pocket, which is the potential opening of the hydrophobic channel of the SMP domain, to be close to the lipid cargos for lipid uptake and/or release.

In summary, together with previous studies, our results lead to a refined model for SMP-mediated lipid transfer, which has important implications for understanding the regulation of lipid homeostasis at MCSs (Fig. 7). At typical ER-PM contact sites (>10 nm in distance), the E-Syt SMP domain, together with the Ca^2+^-bound C2A and C2B domains, shuttles between the tubular ER and the acidic-lipid-enriched PM, and lipids enter and exit via its tip region (Fig. 7a). It is plausible that with the help of C2A domain lipid transfer with low efficiency still occurs at the region where the ER tubule is not extremely curved. This proposed model in which SMP dimer is oriented nearly perpendicular to the ER and PM is also supported by the observed architecture of tricalbin 3 in yeast by cryo-electron tomography (cryo-ET), displaying that its SMP dimer and C2 domains are sequentially arranged in a linear fashion along the axis of its rod-like structure and connect the ER and PM perpendicularly^27^. The shuttling lipid transfer proteins have been suggested to play roles in modulating lipid signaling^56^. Interestingly, the involvement of the acidic-lipid-enriched region in the PM in lipid transfer is compatible with the essential roles of E-Syts in controlling PS exposure and PIP signaling, including autophagy-associated PI3P synthesis in proximity to ER-PM contact sites^30,35,39–41^. We further demonstrate that this PI3P regulation, which contributes to autophagosome formation, requires lipid harboring and membrane association of the SMP domain. The lipid transfer by E-Syts may also be facilitated by autophagy protein VMP1, which is a scramblase and interacts with E-Syts^39,57,58^.

At tight ER-PM contact sites (<10 nm in distance), we propose that the E-Syt SMP dimer acts as a “screw propeller” (Fig. 7b) or a static “tunnel” to transfer lipids between apposed bilayers. According to our in silico MD simulations, as the lipid is difficult to pass through the SMP dimer interface, the “screw propeller” model is preferred, in which one SMP molecule extracts lipids and flips vertically to deliver them to the target membrane. This model is also supported by the results that the membrane association of the SMP domain is weak and unstable. In addition, our in vitro data here provide evidence that the extreme membrane curvature, which is a feature of the spherical ER membrane peaks (<30 nm in diameter) formed in a C2 domain-dependent manner at these regions^26,27,45^, accelerates lipid transfer by the SMP domain. Further elucidating the timescale of membrane binding of SMP domain coupled with lipid extraction by all-atom MD simulations will be important. Moreover, identification of potential partners of E-Syts and tricalbins for regulating the directionality of bulk lipid transfer deserve further investigation, as at cortical ER peaks in yeast the tricalbin SMP domain transfers lipids to repair PM damage upon heat shock^26^. In mammals, E-Syts together with Sec22b-Stx1 promote PM expansion for neuronal development^37^. The ER-PM distance and ER curvature during this process remain to be further explored. Finally, our DNA brick-assisted sorting of vesicles reconstituted with high-molecular-weight proteins and DNA brick-aided lipid transfer system can be adapted to gain further insights into other lipid transfer proteins or enzymes functioning at MCSs.

## Methods

### Reagents

Reagents were obtained from the following sources: Ni Sepharose 6 Fast Flow (Cytiva, 17531802), tris(2-carboxyethyl)phosphine (TCEP, 75259) (Thermo), Optiprep^TM^ Density Gradient Medium (60%, w/v) (Sigma-Aldrich, D1556), glycerol (Sigma-Aldrich, G9012), Anapoe-X-100 (Anatrace, 9002-93-1). All DNA oligonucleotides were purchased from Sangon Biotech. All lipids were obtained from Avanti Polar Lipids: 1-palmitoyl-2-oleoyl-glycero-3-phosphocholine (POPC, 850457); 1-palmitoyl-2-oleoyl-sn-glycero-3-phosphoethanolamine (POPE, 850757); 1-palmitoyl-2-oleoyl-sn-glycero-3-phospho-L-serine (POPS, 840034); L-α-phosphatidylinositol- 4,5-bisphosphate [PI(4,5)P_2_, 840046]; 1,2-dioleoyl-sn-glycero-3-phosphoethanolamine-N-(7-nitro-2-1,3-benzoxadiazol-4-yl) (NBD-PE, 810144); 1,2-dioleoyl-sn-glycero-3-phosphoethanolamine-N-(lissamine rhodamine B sulfonyl) (Rhodamine-PE, 810150); 1,2-dioleoyl-sn-glycero-3-[(N-(5-amino-1-carboxypentyl) iminodiacetic acid) succinyl] [DGS-NTA(Ni), 790404].

### Plasmids

The plasmids encoding E-Syt1_cyto_ (a.a. 93–1104, pCMV6-AN-His), SMP-C2AB (a.a. 93-634, pCMV6-AN-His), SMP-C2AB K227E (a.a. 93–634, pCMV6-AN-His), SMP of E-Syt1 (a.a. 134–327, pET-28a), EGFP-E-Syt3 (a.a. 1–886, pEGFP-C1), mCherry-ORP5 (a.a. 1–879, pmCherry-C1) and mCherry-LC3 (pmCherry-C1) were described previously^10,30,31,52^. The region coding the full-length E-Syt1 (a.a. 1–1104) was cloned into the pCMV6-AN-His vector using the AscI and NotI sites. The region coding the SMP of E-Syt2 (a.a. 162–355) and the SMP of E-Syt3 (a.a. 113–304) were cloned into the pET-28a vector using the NheI and XhoI sites. Point mutations were introduced by site-directed mutagenesis. STIM-E-Syt3 (signal peptide and transmembrane domain of STIM1 and a.a. 71-886 of E-Syt3) was generated by overlap PCR and cloned into the pEGFP-N1 vector.

### Protein expression and purification

Full-length E-Syt1 was expressed in Expi293 cells (Thermo Fisher Scientific, A14527). Cells were harvested by centrifugation, washed twice with buffer A [25 mM Hepes, pH 7.4, 300 mM NaCl, 1× complete EDTA-free protease inhibitor cocktail (Roche), 0.5 mM TCEP], and lysed by three freeze-thaw cycles using liquid nitrogen. Membranes were pelleted by centrifugation at 200,000 × *g* for 1 h at 4 °C using a SW 41 rotor (Beckman Coulter) and subsequently solubilized in 2.5% (w/v) Anapoe X-100 in buffer A for 1 h at 4 °C. The extract was centrifuged at 200,000 × *g* for 1 h at 4 °C using a SW 41 rotor and the protein was purified from the supernatant by an Ni-NTA column. After elution using 0.1% (w/v) Anapoe X-100 and 200 mM imidazole in buffer A, the protein was passed through a desalting column with 0.1% (w/v) Anapoe X-100 in buffer A to remove the imidazole. The purified E-Syt1 was mixed with liposomes at a protein to lipid ratio of 1:500 at 4 °C for 1 h with gentle shaking. The final Anapoe X-100 concentration was added to 0.1% (w/v) and lipid concentration was 1 mM. The reconstitution of proteins into liposomes was achieved by removing the detergent with bio-beads SM-2 resin (Bio-Rad).

Soluble fragments of E-Syts were expressed in Expi293 cells or Rosetta (DE3) (Biomed) *E. coli* cells and purified as described previously^30^. Briefly, cells were harvested and lysed in buffer A by three freeze-thaw cycles using liquid nitrogen (for Expi293 cells) or by sonication (for bacteria). The suspension was clarified by centrifugation at 200,000 × *g* for 1 h at 4 °C using a TYPE 45 Ti rotor. The protein was isolated by a Ni-NTA column and further purified by gel filtration in buffer A. Fractions containing E-Syt1 fragments were pooled and concentrated.

### Liposome preparation

ER-like donor liposomes were composed as follows: 87:10:1.5:1.5 mole percent of POPC: POPE: NBD-PE: Rhodamine-PE or 77:10:1.5:1.5:10 mole percent of POPC: POPE: NBD-PE: Rhodamine-PE: DGS-NTA(Ni). PM-like acceptor liposomes were composed as follows: 85:10:5 mole percent of POPC: POPS: PI(4,5)P_2_ or 70:20:10 mole percent of POPC: POPS: PI(4,5)P_2_. ER-like acceptor liposomes were composed as follows: 90:10 mole percent of POPC: POPE. PM-like donor liposomes were composed as follows: 82:10:5:1.5:1.5 mole percent of POPC: POPS: PI(4,5)P_2_: NBD-PE: Rhodamine-PE. Liposome preparation was performed as described previously^30^. Briefly, lipid mixtures were dried with N_2_ to form a film. Buffer A was added to the tube to rehydrate the lipid films, and the suspension underwent ten freeze-thawing cycles using liquid nitrogen. Extruded liposomes were formed by extrusion through polycarbonate filters with a pore size of 30, 100, 400, or 800 nm (Avanti Polar Lipids). Sonicated liposomes were formed by sonication for 10 min with 1 s sonication on and 1 s pulse using a probe sonicator.

### DNA brick preparation

The DNA brick was assembled as described previously^47^. Briefly, the unmodified and cholesterol-modified oligonucleotides were mixed in buffer B (25 mM Hepes, pH 7.0, 400 mM KCl, 10 mM MgCl_2_) and assembled to form DNA bricks using thermal annealing from 95 to 4 °C (held at 95, 65, 50, 42, 37, 22, and 4 °C for 5 min each). The assembled DNA bricks were placed on top of a 5–20% glycerol gradient. The sample-loaded density medium was centrifuged at 205,000 × *g* for 4.5 h at 4 °C using a TLS 55 rotor (Beckman Coulter) and analyzed by agarose gel electrophoresis. Fractions containing well-folded DNA bricks were combined and concentrated. The purified products were stored at −20 °C.

### DNA-brick-assisted liposome sorting

The DNA bricks and liposomes or proteoliposomes were mixed at a DNA brick to lipid ratio of 1:375 and incubated at 4 °C over night with gentle shaking. The DNA brick-coated liposomes or proteoliposomes were subjected to centrifugation in iodixanol gradients. A quasi-linear gradient containing 0–18% (w/v) iodixanol was loaded on top of sample containing 22.5% iodixanol. Gradients were then centrifuged at 215,000 × *g* for 4.5 h at 4 °C using a SW 55 Ti rotor (Beckman Coulter). After ultracentrifugation, the content of a tube was fractioned from top to bottom. The fractions were examined by negative-staining TEM and those containing liposomes or proteoliposomes were treated with DNase I (Thermo) to digest the DNA bricks. To remove iodixanol, the liposomes or proteoliposomes were concentrated by centrifugation at 10,000 × *g* at 4 °C on Amicon filtration units with 30 kD NMWL, and the concentrated samples were diluted in buffer B and concentrated again for a total of five times. To determine the protein to lipid ratio, the concentration of protein in each fraction was assessed by the density of the corresponding protein band on SDS-PAGE gel stained with Coomassie Blue using BSA concentration as standards, and lipid concentrations were measured by Rhodamine-PE absorbance at 574 nm, which was 1.5% of total lipids.

### Transmission electron microscopy

To prepare negatively stained liposomes or proteolipsomes with or without DNA bricks, a drop of sample (5 μL) was deposited on a glow discharged formvar/carbon-coated copper grid and incubated for 1–3 min at room temperature. Fluid was then blotted away. The grid was immediately stained for 3 min with 2% (w/v) uranyl formate. Grids were examined using a JEOL JEM-1400 Plus microscope (acceleration voltage: 80 kV). Images were acquired by an Advanced Microscopy Technologies bottom-mount 4k × 3k charge-coupled device camera using the AMT Image Capture Engine. Liposome sizes were measured from electron micrographs using ImageJ (NIH) as described previously^47^.

### Lipid transfer assays

All in vitro lipid transfer assays were performed as described previously^35^. Briefly, reactions were performed in 100 μL volumes. The final lipid concentration was 0.5 mM with donor and acceptor liposomes added at a 1:1 ratio. The reaction buffer was 25 mM Hepes, pH 7.4, 150 mM NaCl, 0.5 mM TCEP. Reactions were initiated by the addition of proteins or proteoliposomes to the mixtures (protein: lipid ratio of 1: 1000) in a 96-well plate (Corning). The fluorescence intensity of NBD was monitored at an excitation of 460 nm and emission of 538 nm every 10 or 30 s over 10 or 30 min at room temperature or 37 °C using a Cytation 5 Imaging Reader (BioTek). All data were corrected by setting the data point at 0 min to zero and subtracting the baseline values obtained at 0 min. The data were expressed as a percentage of the maximum fluorescence determined after adding 10 μL of 2.5% dodecylmaltoside (Avanti Polar Lipids) to the reactions after 10 or 30 min. The slope of the initial linear portion (after the rapid uptake phase) of the lipid transfer curve was calculated to determine the initial rate.

### Liposome tethering assays

Liposome tethering assays were performed as described previously^30^. Briefly, the reaction conditions were same as the lipid transfer assays. The reactions were initiated by the addition of proteins or proteoliposomes to the mixture of liposomes in a 96-well plate (Corning). The absorbance at 405 nm was measured to assess turbidity every 10 s over 10 min at room temperature using a Cytation 5 Imaging Reader (BioTek). Data were expressed as absolute absorbance values subtracted by the absorbance at 0 min.

### Lipid harboring assays

Purified WT or mutated SMP-C2AB of E-Syt1 (19 μL at 40 μM) was mixed with or without 1 μL NBD-PE or a mixture of NBD-PE and POPC (1:10 ratio) in methanol and incubated at 4 °C for 1 h. The fluorescence of NBD-PE and Coomassie-stained proteins were visualized on a native PAGE gel.

### Liposome sedimentation assays

A total of 2 μM protein was incubated with liposomes (protein to lipid ratio of 1: 500) in buffer containing 25 mM Hepes, pH 7.4, 150 mM NaCl, 0.5 mM TCEP for 1 h at room temperature, followed by ultracentrifugation at 16,100 × *g* for 1 h at 4 °C. The membrane pellets were re-suspended in the same buffer. Equal volumes of supernatants and pellets were run on SDS-PAGE and stained with Coomassie Blue.

### Molecular dynamics simulations

The crystal structure of E-Syt2^28^ was used to model the dimeric SMP (SMP_2_). The coarse-grained (CG) model of SMP_2_ used the most recent development version of the Martini 3 force field^50^. The CG model of SMP_2_ was prepared using the martinize.py program and subsequently embedded in an ER-like membrane model consisting of 80% POPC:20% POPE and a PM-like model consisting of 85% POPC:10% POPS:5% PI(4,5)P_2_ with dimensions of 12 × 12 × 18 nm^3^ (Table S2) using the INSANE protocol^59^. The mutants Y257A, F258A and R256E were prepared using martinize.py. The ElNeDyn elastic-network approach was employed to restrain the protein structure, using a force constant of 1000 kJ/mol/nm^2^ and the lower and upper limits of the cutoff distance of 0.5 and 0.9 nm, respectively. The systems were solvated using a CG Martini water model and neutralized by adding NaCl at a concentration of 0.15 M to mimic physiological conditions. To alleviate the dependence on the initial orientation of SMP, the principal axis of the SMP dimer was set to be either parallel or vertical to the normal of the lipid bilayers and 2.5 nm away from the membrane surface, resulting in two different initial conformations corresponding to the lying-down and standing-up models (Table S2). Note that a model without the membrane was also built as a control.

First, the CG system was minimized for 5000 steps with the steepest descent method and subsequently equilibrated by following the standard CHARMMGUI equilibrium protocol^60^. Finally, each production run was performed for 10 μs in the semi-isotropic NPT ensemble using a time step of 20 fs. The temperature of the system was kept at 310 K with the velocity rescaling thermostat. The pressure was kept at 1 bar using the Parrinello-Rahman barostat with a compressibility of 3 × 10^−4^ bar^−1^ and a coupling constant of 12 ps.

For the all-atom (AA) MD simulations, we used the CHARMM36m force field for the protein and lipids and prepared the systems using the CHARMM-GUI server. To explore lipid sliding along the SMP domain, we replaced one of the lipids in the crystal structure of E-Syt2 with a POPE lipid and removed the other lipids. To model the membrane binding conformation of the SMP domain, we inserted the SMP dimer in a mixed 80% POPC:20% POPE bilayer based on the insertion depth obtained from the CG simulations.

The box size was 5.9 × 5.9 × 10.9 nm^3^ and 12.4 × 12.4 × 14.9 nm^3^ for the lipid sliding model and SMP insertion model, respectively. The systems were solvated using the TIP3P solvent model. Periodic boundary conditions were employed, and the particle-mesh Ewald method was used for the treatment of long-range electrostatic interactions. The simulations were conducted at a constant semi-isotropic pressure of 1 atm and a temperature of 310 K using the Parrinello–Rahman barostat and the Nosé–Hoover thermostat, respectively.

To check whether the loaded lipid can move along the hydrophobic channel of the SMP dimer in a reasonable MD time, we added an additional ratchet-and-pawl-like potential to the center of mass of the headgroup of the bound POPE lipid with a force constant of 1 kJ/(mol nm^2^) using the adiabatic bias molecular dynamics (ABMD) method^61,62^. The biasing potential is zero when the lipid moves to the other tip but provides a penalty as it moves back, so that we were able to accelerate its motion from one tip to the other. The ABMD simulation was run for 50 ns.

All simulations were performed with GROMACS (version 2021.5). Trajectories were saved every 1 ns. The results were analyzed with PLUMED^63^, VMD^64^ and in-house scripts.

### Cell culture and transfection

HeLa cells (ATCC, CCL-2) were cultured in Dulbecco’s modified Eagle medium (DMEM, Gibco) supplemented with 100 U/ml penicillin, 0.1 mg/ml streptomycin, and 10% fetal bovine serum (FBS, Biological Industries) at 37 °C under 5% CO_2_. Cells were transfected with plasmids using Polyethylenimine Linear (PEI, Yeasen Biotechnology) according to the manufacturer’s instructions.

### Fluorescence microscopy

Cells were grown to 60% confluence on a 14-mm coverslip, washed twice with PBS, and fixed with freshly prepared 4% formaldehyde at 37 °C for 15 min. Fixed cells were imaged using a laser scanning confocal microscope (Zeiss LSM 800 with Airyscan) with a 63× oil-immersion objective. EGFP was excited by a 488 nm laser and fluorescence was detected within the wavelength range of 490–575 nm. RFP was excited by a 568 nm laser and fluorescence was detected within the wavelength range of 570–700 nm. The number of LC3 puncta was determined with ImageJ (NIH) and analyzed by manually drawing regions at edges of cells.

### Statistical analysis

No statistical method was used to predetermine sample size. For fluorescence microscopy using cultured cells, values were obtained from three independent experiments. Data were compared by either the two-tailed Student’s t-test or the one-way or two-way ANOVA with Bonferroni’s or Sidak’s multiple comparisons test as appropriate with Prism 8 (GraphPad software).

### Reporting summary

Further information on research design is available in the Nature Portfolio Reporting Summary linked to this article.

## Supplementary information

Supplementary Information

Description of Additional Supplementary Files

Supplementary Movie 1

Reporting Summary

## Data Availability

All data are available from the corresponding authors upon request. The source data underlying Figs. 1b, 1d, 1g, 2b–d, 3a–b, 3d–e, 4b, c, 5g–m, 6b and Supplementary Fig 1b–d, 2d, e, 3a, 4, 5a, 7, 8, 10a are provided as a Source Data file. The crystal structure of SMP domain of human E-Syt2 has previously been deposited in the Protein Data Bank (PDB) under accession code 4P42 [10.2210/pdb4P42/pdb]. Supplementary information Source data are provided with this paper.

## Source data

Source Data
