# Supplementary material for: Insights into membrane association of the SMP domain of extended synaptotagmin
Source: Nat Commun. 2023 Mar 17;14:1504. doi: 10.1038/s41467-023-37202-8 (PMC10023780; doi:10.1038/s41467-023-37202-8)
Supplement: Supplementary file 1 — Supplementary Information [file 41467_2023_37202_MOESM1_ESM.pdf]

Supplementary Information for

Insights into membrane association of the SMP domain of extended synaptotagmin

Yunyun Wang[1], Zhenni Li[1], Xinyu Wang[2], Ziyuan Zhao[2], Li Jiao[2], Ruming Liu[2], Keying Wang[3], Rui Ma[4], Yang Yang[5], Guo Chen[1], Yong Wang[3,6,*], Xin Bian[1,*]

[1]State Key Laboratory of Medicinal Chemical Biology, College of Life Sciences, Frontiers Science Center for Cell Responses, Nankai University, Tianjin 300071, China;

[2]College of Life Sciences, Nankai University, Tianjin 300071, China;

[3]College of Life Sciences, Zhejiang University, Hangzhou 310027, China;

[4]College of Physical Science and Technology, Xiamen University, Xiamen 361005, China;

[5]Institute of Molecular Medicine, Renji Hospital, School of Medicine, Shanghai Jiao Tong University, Shanghai 200127, China;

[6]The Provincial International Science and Technology Cooperation Base on Engineering Biology, International Campus of Zhejiang University, Haining, 314400, China;

[*]Correspondence: xin.bian@nankai.edu.cn
yongwang_isb@zju.edu.cn

This PDF file includes:

Supplementary Figures 1-10 and Supplementary Tables 1-2

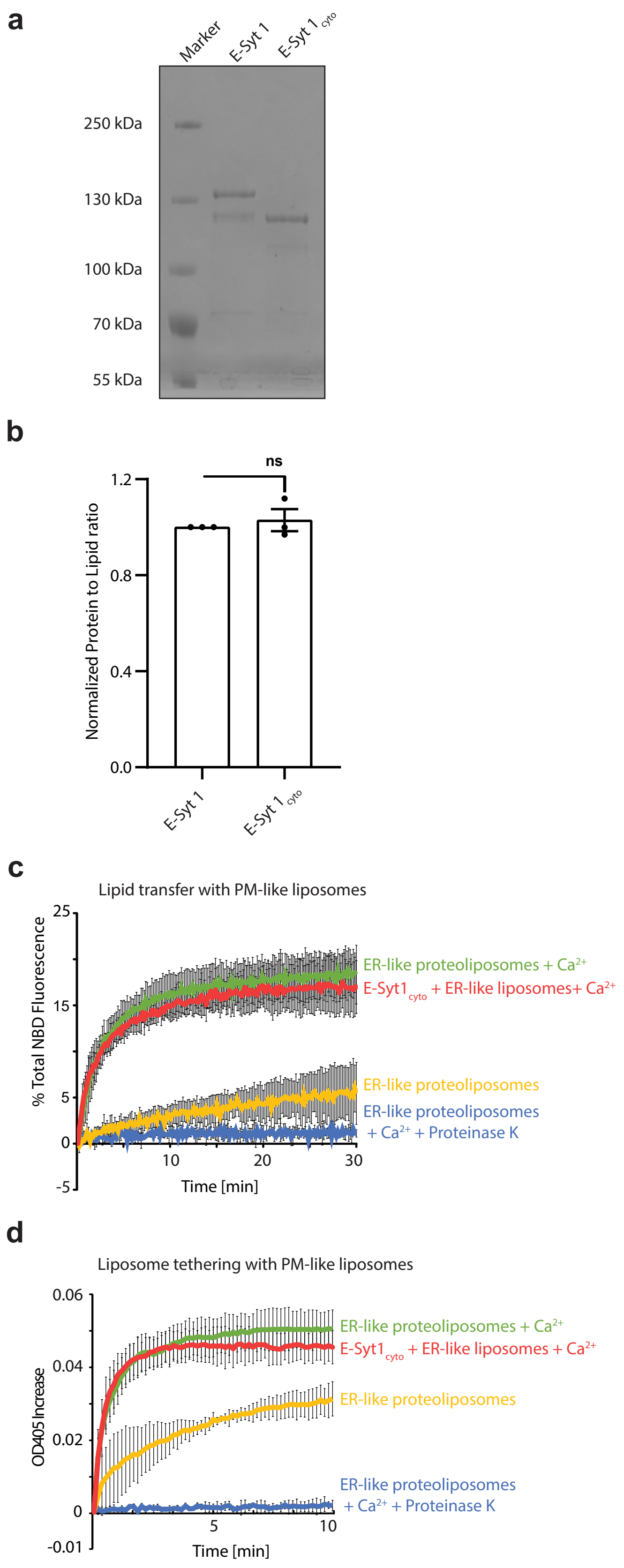

Supplementary Fig. 1 Lipid transfer and membrane tethering mediated by full-length E-Syt1 *in vitro*. a, Purified E-Syt1 and E-Syt1$_{cyto}$ were run on SDS-PAGE and stained with Coomassie blue. **b,** The proteoliposomes containing E-Syt1 and E-Syt1$_{cyto}$ were run on SDS-PAGE and stained with Coomassie blue. Quantifications of the ratios of the protein levels determined by densitometry (ImageJ) to the lipid levels determined by Rhodamine-PE fluorescence are shown as mean ± SD (n = 3 independent experiments). ns, not significant by two-tailed Student's t-tests. **c,** Time courses of lipid transfer between ER-like donor proteoliposomes containing E-Syt1 or liposomes with E-Syt1$_{cyto}$ and PM-like acceptor liposomes in the presence of Ca^{2+} or proteinase K at room temperature as assessed by dequenching of NBD-PE fluorescence (mean ± SD, n = 3 independent experiments). **d,** Time courses of the tethering of ER-like donor proteoliposomes containing E-Syt1 or liposomes with E-Syt1$_{cyto}$ and PM-like acceptor liposomes in the presence of Ca^{2+} or proteinase K at room temperature as assessed by an increase in turbidity (OD at 405 nm). Data are presented as mean ± SD (n = 3 independent experiments). Source data are provided as a Source Data file.

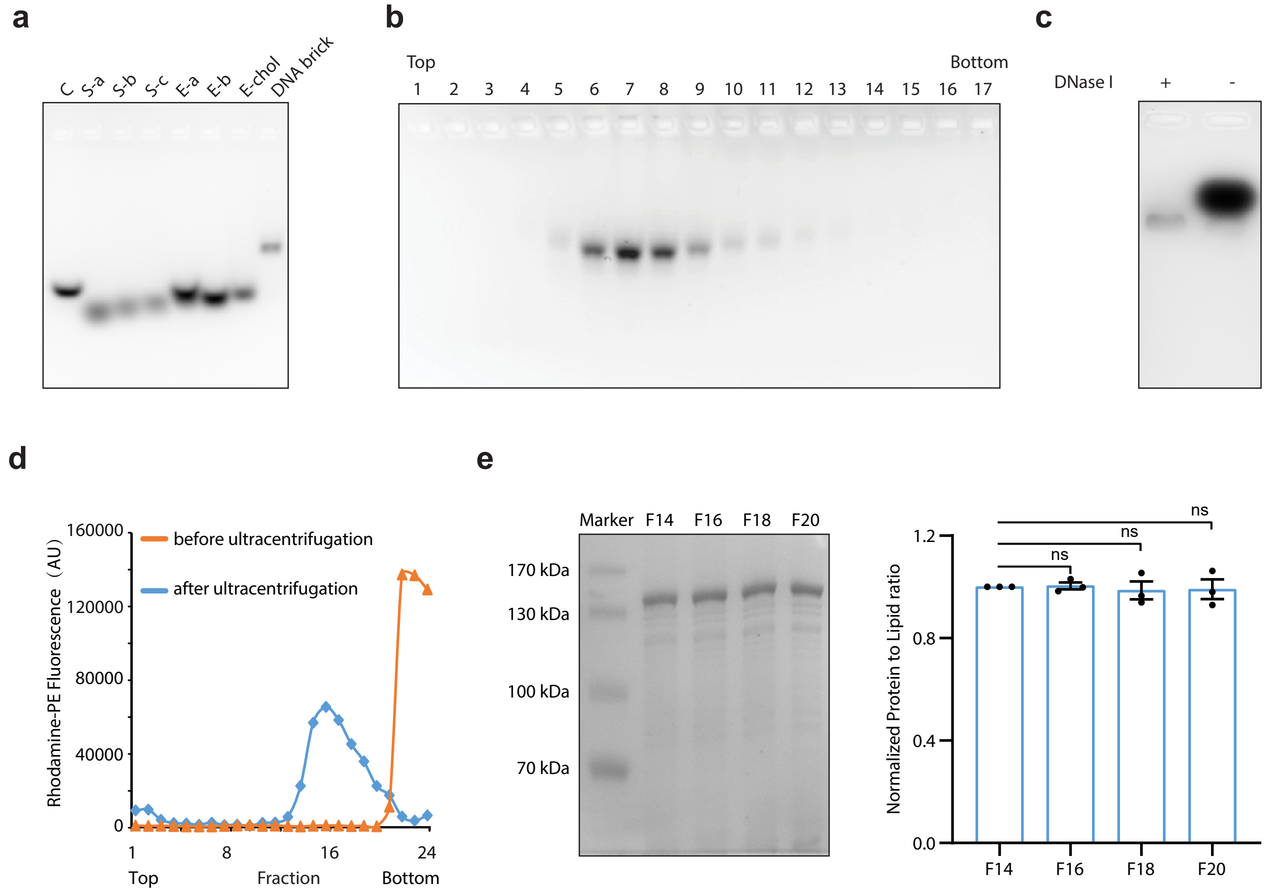

Supplementary Fig. 2 DNA brick-assisted proteoliposome sorting. a, Seven oligonucleotides and self-assembled DNA bricks were analyzed by agarose gel electrophoresis. This experiment was repeated five times with similar results. **b,** DNA bricks were purified by rate-zonal centrifugation, and fractions were analyzed by agarose gel electrophoresis. This experiment was repeated five times with similar results. **c,** DNA bricks were incubated with liposomes composed of 90% POPC and 10% POPE at a DNA brick to lipid ratio of 1: 375 at 4 °C over night followed by DNase I treatment or not for 1 h at 37 °C. The samples were run on SDS-agarose gel electrophoresis and analyzed by SYBR Gold staining. The SDS caused the lysis of DNA brick-coated liposomes in the gel. This experiment was repeated five times with similar results. **d,** Plot showing the distribution of fluorescence of Rhodamine-PE in the fraction collected from top (F1) to the bottom (F24) of the gradient before (red curve) or after (blue curve) ultracentrifugation. Experiments were repeated independently five times with similar results. **e,** Left, proteoliposomes in F14, F16, F18 and F20 were sedimented and the pellets were run on SDS-PAGE and stained with Coomassie blue. Right, quantifications of the ratios of the protein levels determined by densitometry (ImageJ) to the lipid levels determined by Rhodamine-PE fluorescence are shown as mean ± SD (n = 3 independent experiments). ns, not significant by one-way ANOVA with Bonferroni's multiple comparisons test. Source data are provided as a Source Data file.

a

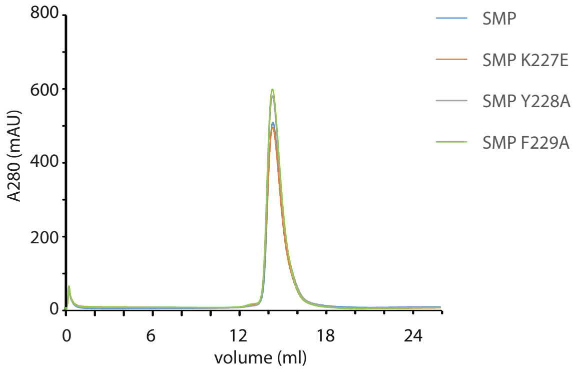

b

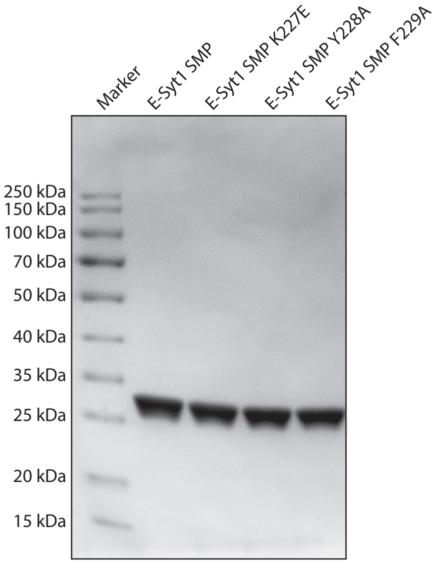

Supplementary Fig. 3 Purity of WT and mutated SMP domain used in this study. a, Purified WT and mutated SMP domain were subjected to gel filtration on a Superdex 200 Increase 10/300 GL column. **b,** Purified WT and mutated SMP domain were run on SDS-PAGE and stained with Coomassie blue. Experiments were repeated independently five times with similar results. Source data are provided as a Source Data file.

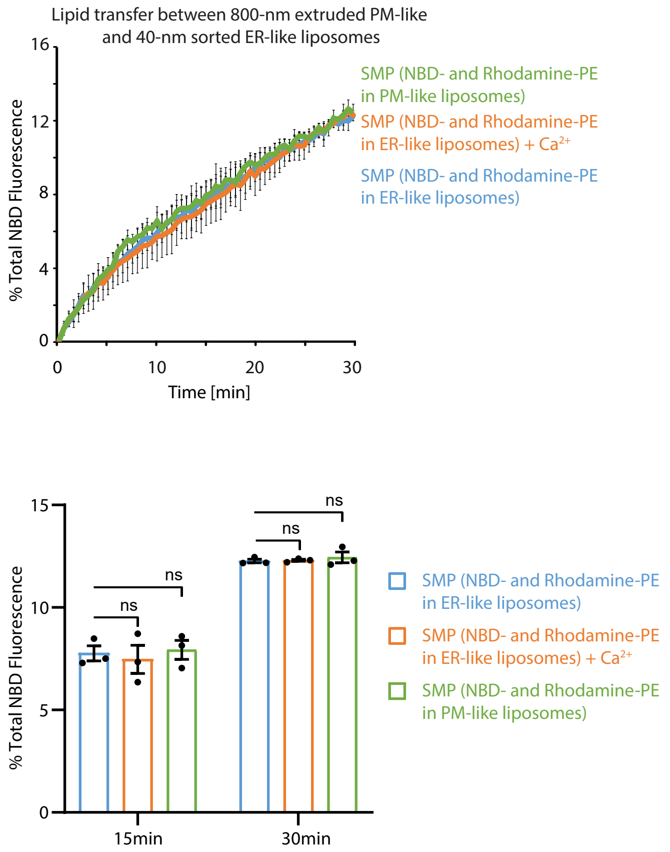

Supplementary Fig. 4 SMP-mediated lipid transfer is bidirectional and independent on Ca^{2+}. Top, Time courses of SMP-mediated lipid transfer between ER-like and PM-like liposomes in the absence or presence of Ca^{2+} at 37 °C as assessed by dequenching of NBD-PE fluorescence. Bottom, quantifications of NBD fluorescence after incubation for 15 min or 30 min. Data are presented as mean ± SD (n = 3 independent experiments). ns, not significant by two-way ANOVA with Bonferroni's multiple comparisons test. Source data are provided as a Source Data file.

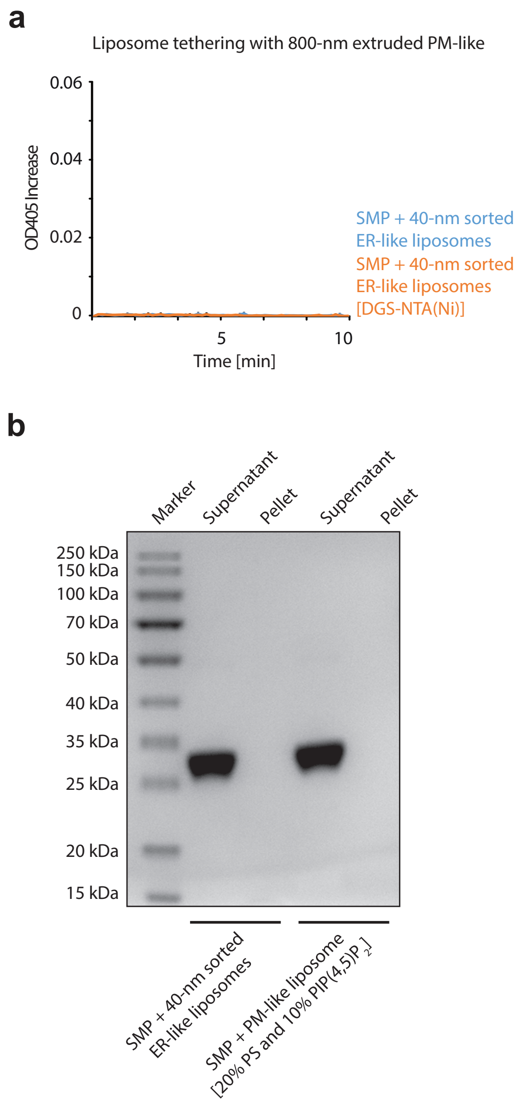

Supplementary Fig. 5 SMP does not stably associate with the membranes. a, Time courses of the tethering of ER-like donor liposomes containing DGS-NTA(Ni) or not and PM-like acceptor liposomes in the presence of SMP at 37 °C as assessed by an increase in turbidity (OD at 405 nm). Data are presented as mean ± SD (n = 3 independent experiments). **b,** Purified SMP domain was incubated with 40-nm sorted ER-like liposomes or PM-like liposomes containing 70% PC, 20% PS and 10% PI(4,5)P_2. Liposomes were sedimented and supernatant and pellet were run on SDS-PAGE and analyzed by Coomassie blue staining. This experiment was repeated three times with similar results. Source data are provided as a Source Data file.

```
                         ★                                                       ★★●●
BtE-Syt1 SMP  172  ...AVRGSNPHLQTFTFTRVELGEKPLRILGVKVHTGQ-SKKQILLDLNISYVGDLQIDVEVKKYFCKAGVKGMQLH...
DrE-Syt1 SMP  152  ...SIRATSAHLQTLSFTKVDLGDRAMKVVGVKAYTEF-DRRQVILDLYISYAGDVEINVEVKKYFCKAGVKGIQLH...
MmE-Syt1 SMP  158  ...AVRGANPHLQTFTFTRVELGEKPLRIIGVKVHPSQ-RKDQILLDLNVSYVGDVQIDVEVKKYFCKAGVKGMQLH...
OcE-Syt1 SMP  155  ...AVRGSNPHLQTFTFTRVELGEKPLRVIGVKVHPSQ-RKDQILLDLNVSYVGDVQIDVEVKKYFCKAGVKGMQLH...
XtE-Syt1 SMP  135  ...TIRASNTHLSTFYFTKINVGEKAPKVTGVKAHTEF-DKKQIILDLHLSYVGDIEVNVEVKKYFCKAGIKGMQLH...
HsE-Syt1 SMP  168  ...AVRGSNPHLQTFTFTRVELGEKPLRIIGVKVHPGQ-RKEQILLDLNISYVGDVQIDVEVKKYFCKAGVKGMQLH...
HsE-Syt2 SMP  196  ...AVRGANTHLSTFSFTKVDVGQQPLRINGVKVYTENVDKRQIILDLQISFVGNCEIDLEIKRYFCRAGVKSIQIH...
HsE-Syt3 SMP  147  ...KIREKSIHLRTFTFTKLYFGQKCPRVNGVKAHTNTCNRRRVTVDLQICYIGDCEISVELQK-I-QAGVNGIQLQ...

                         ■                     ■■
BtE-Syt1 SMP  245  ...GVLRVILEPLMGDLPIVGAVSMFFIRRPTLDINWTGMTNLLDIPGLSSLSDTMIMDSIAAFLVLPNRLLVPLVP...
DrE-Syt1 SMP  225  ...GKLRVILEPLIGDVPLVGAITMFFIRRPKLDINWTGMTNLLDIPGLNAMSDTMIMDAIASFLVLPNRLTVPLVA...
MmE-Syt1 SMP  231  ...GVLRVILEPLTGDLPIVGAVSMFFIKRPTLDINWTGMTNLLDIPGLSSLSDTMIMDSIAAFLVLPNRLLVPLVP...
OcE-Syt1 SMP  228  ...GVLRVILEPLMGDVPIVGAVSMFFIRRPTLDINWTGMTNLLDIPGLSSLSDTMIMDSIAAFLVLPNRLLVPLVP...
XtE-Syt1 SMP  208  ...GMLRVILEPLIGDVPIVGAMTLFFIRRPVLDINWTGLTNLLDIPGLNLMSDTMVMDIISGFLVLPNRLAIPLAS...
HsE-Syt1 SMP  241  ...GVLRVILEPLIGDLPFVGAVSMFFIRRPTLDINWTGMTNLLDIPGLSSLSDTMIMDSIAAFLVLPNRLLVPLVP...
HsE-Syt2 SMP  270  ...GTMRVILEPLIGDMPLVGALSIFFLRKPLLEINWTGLTNLLDVPGLNGLSDTIILDIISNYLVLPNRITVPLVS...
HsE-Syt3 SMP  219  ...GTLRVILEPLLVDKPFVGAVTVFFLQKPHLQINWTGLTNLLDAPGINDVSDSLLEDLIATHLVLPNRVTVPVKK...
```

Supplementary Fig. 6 Sequence alignment of basic patches of E-Syt SMP domains from various species. *B. taurus* E-Syt1 (BtE-Syt1), *D. rerio* E-Syt1 (DrE-Syt1), *M. musculus* E-Syt1 (MmE-Syt1), *O. cuniculus* E-Syt1 (OcE-Syt1), *X. tropicalis* E-Syt1 (XtE-Syt1), *H. sapiens* E-Syt1 (HsE-Syt1), *H. sapiens* E-Syt2 (HsE-Syt2), *H. sapiens* E-Syt3 (HsE-Syt3). The positively charged residues at the side region (cyan squares) and the positively charged residues (blue stars) and large hydrophobic residues (magenta circles) at the tip region in the crystal structure of human E-Syt2 are indicated.

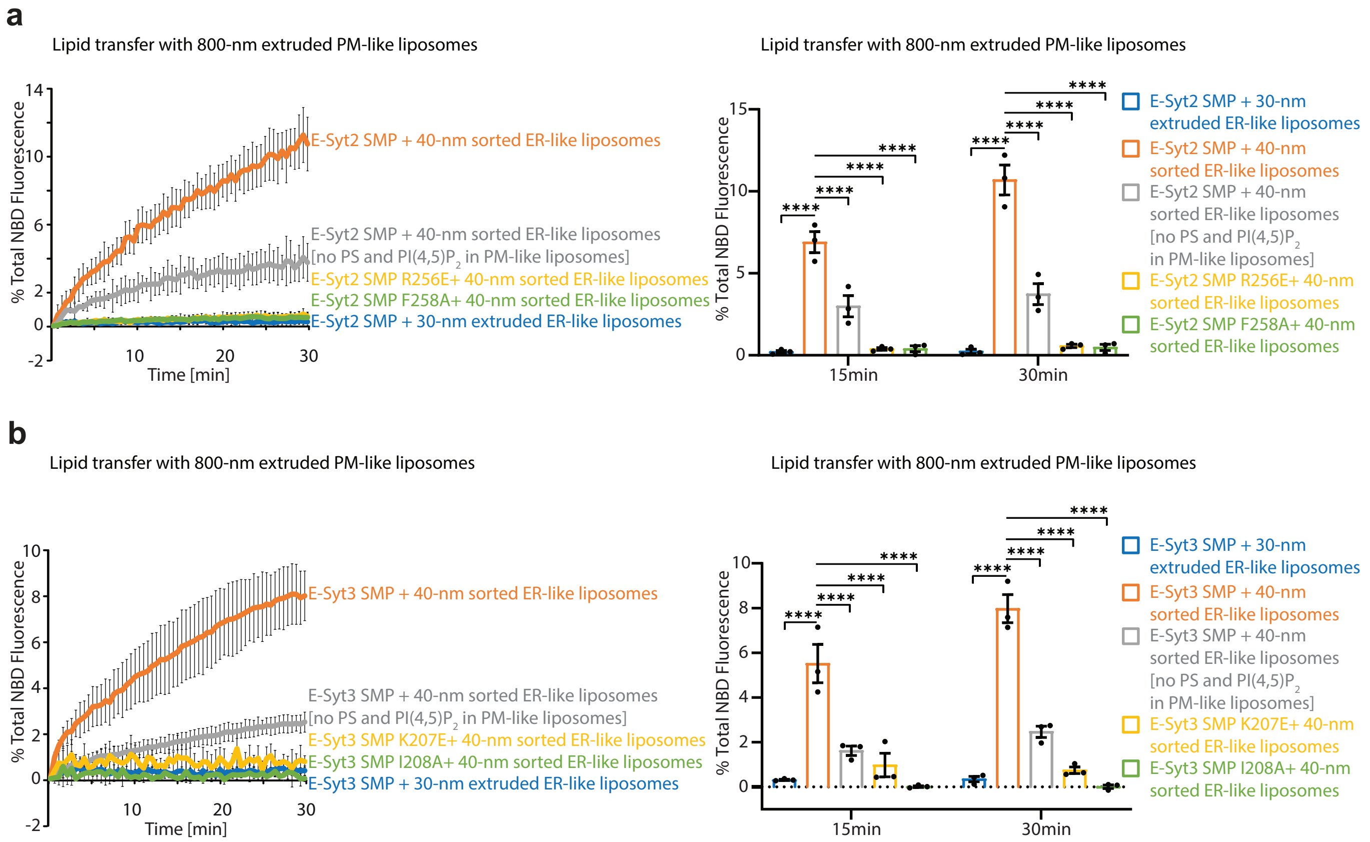

Supplementary Fig. 7 Lipid transfer by SMP domains of E-Syt2 and E-Syt3. a and b, Top, time courses of lipid transfer between distinctly sized ER-like donor liposomes and PM-like acceptor liposomes with or without $PI(4,5)P_2$ and PS in the presence of WT or mutated SMP of E-Syt2 (a) or E-Syt3 (b) at 37 °C as assessed by dequenching of NBD-PE fluorescence. Bottom, Bottom, quantifications of NBD fluorescence after incubation for 15 min or 30 min. Data are presented as mean ± SD (n = 3 independent experiments). **** P<0.0001 by two-way ANOVA with Bonferroni's multiple comparisons test. P values (a, 15min): $2.6x10^{-8}$ [(E-Syt2 SMP + 30-nm extruded ER-like liposomes) vs (E-Syt2 SMP + 40-nm sorted ER-like liposomes)], $9.0x10^{-5}$ [(E-Syt2 SMP) vs (E-Syt2 SMP no PS and PIP_2 in PM-like liposomes)], $4.1x10^{-8}$ [(E-Syt2 SMP) vs (E-Syt2 SMP R256E)], $4.2x10^{-8}$ [(E-Syt2 SMP) vs (E-Syt2 SMP F258A)]; (a, 30 min): $9.0x10^{-12}$ [(E-Syt2 SMP + 30-nm extruded ER-like liposomes) vs (E-Syt2 SMP + 40-nm sorted ER-like liposomes)], $1.3x10^{-8}$ [(E-Syt2 SMP) vs (E-Syt2 SMP no PS and PIP_2 in PM-like liposomes)], $1.6x10^{-11}$ [(E-Syt2 SMP) vs (E-Syt2 SMP R256E)], $1.4x10^{-11}$ [(E-Syt2 SMP) vs (E-Syt2 SMP F258A)]; (b, 15 min): $1.0x10^{-7}$ [(E-Syt3 SMP + 30-nm extruded ER-like liposomes) vs (E-Syt3 SMP + 40-nm sorted ER-like liposomes)], $8.7x10^{-6}$ [(E-Syt3 SMP) vs (E-Syt3 SMP no PS and PIP_2 in PM-like liposomes)], $8.9x10^{-7}$ [(E-Syt3 SMP) vs (E-Syt3 SMP K207E)], $4.0x10^{-8}$ [(E-Syt3 SMP) vs (E-Syt3 SMP I208A)]; (b, 30 min): $1.3x10^{-10}$ [(E-Syt3 SMP + 30-nm extruded ER-like liposomes) vs (E-Syt3 SMP + 40-nm sorted ER-like liposomes)], $3.9x10^{-8}$ [(E-Syt3 SMP) vs (E-Syt3 SMP no PS and PIP_2 in PM-like liposomes)], $3.4x10^{-10}$ [(E-Syt3 SMP) vs (E-Syt3 SMP K207E)], $5.9x10^{-11}$ [(E-Syt3 SMP) vs (E-Syt3 SMP I208A)]. Source data are provided as a Source Data file.

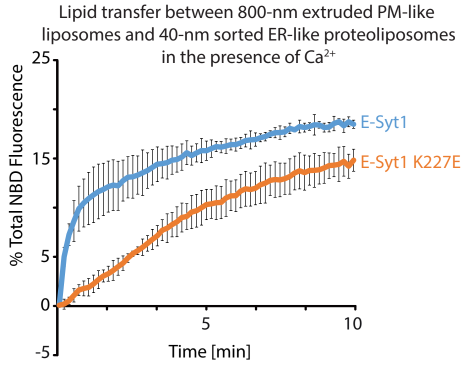

Supplementary Fig. 8 E-Syt1-dependent lipid transfer is facilitated by membrane association of the tip region of the SMP domain. Time courses of lipid transfer between ER-like donor proteoliposomes containing WT or mutated E-Syt1 and PM-like acceptor liposomes in the presence of Ca^{2+} at room temperature as assessed by dequenching of NBD-PE fluorescence (mean ± SD, n = 3 independent experiments). Source data are provided as a Source Data file.

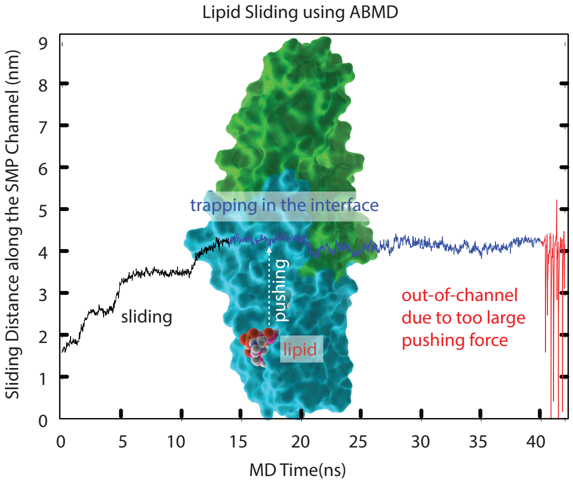

Supplementary Fig. 9 There is a huge energy barrier for lipid sliding at the interface of the SMP dimer. The lipid sliding trajectory of an adiabatic bias molecular dynamics (ABMD) simulation in which a ratchet-and-pawl-like potential was added on the center of mass of the headgroup of the bound POPE lipid to accelerate its motion from one tip to the other. The initial structure of the lipid-bound SMP dimer is shown. The headgroup of the bound POPE is shown as spheres, and the two SMP monomers are colored in green and cyan, respectively. The direction of the biasing force is illustrated by a dashed white arrow. The trajectory showed that the loaded lipid was sliding along the hydrophobic groove of one SMP in the first 15 ns (black line), and was subsequently trapped in the SMP dimer interface (blue line) even under a continuing driving force, and finally dropped off from the binding pocket of SMP after 40 ns, thus resulting in free diffusion in the solvent (red line).

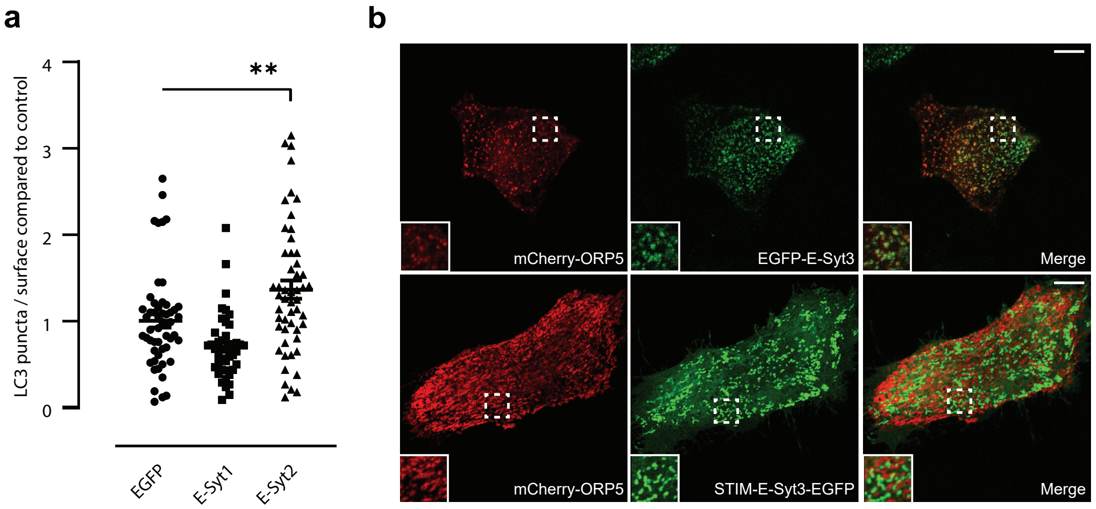

Supplementary Fig. 10 E-Syt2 and E-Syt3 are required for autophagosome biogenesis. a, Quantification of LC3 puncta per area compared to control. Data are presented as mean ± SEM (n = 53, 43, and 52 cells from left to right). ** P<0.01 by one-way ANOVA with Bonferroni's multiple comparisons test. P values: $6.9x10^{-3}$ [(EGFP) vs (E-Syt2)]. **b,** Confocal images of HeLa cells co-expressing mCherry-ORP5 and EGFP-E-Syt3 (top) or STIM-E-Syt3-EGFP (bottom). Insets show enlarged areas framed by dotted boxes. Scale bar, 10 μm. This experiment was repeated three times with similar results. Source data are provided as a Source Data file.

Supplementary Table 1. Sequences of unmodified and cholesterol-modified oligonucleotides.

Name	Sequence
C	AGGCATATTGAATCGTTTACAGGATTAGTAATTAACAGCTTTAATATCATCGCCCATCGTAGGTTTCTTGCC
S-a	GACGACAGAGGTTGCTAGGCG
S-b	TTACCGTGTGTGTTAAGGTGG
S-c	ACCGAGCCTCCGTCAACATCG
E-a	CCACCTTAACACGCGATGATATTGCTGTTAATTAGGCTCGGT
E-b	CGATGTTGACGGACTAATCCTGTCGATTCAATATCTGTCGTC
E-Chol	CGCCTAGCAACCTGCCTGGCAAGCCTACGATGGACACGGTAA/3CholTEG/

Supplementary Table 2. MD simulations of the SMP domain of E-Syt2.

Model	Membrane	Force Field	MD Timescale	Note
Lying-down SMP	80% PC:20% PE	Martini3 (CG)	10 µs x 2	unbiased
	85% PC:10% PS:5%PIP_2	Martini3 (CG)	10 µs x 2	unbiased
Standing-up SMP	80% PC:20% PE	Martini3 (CG)	10 µs x 2	unbiased
	85% PC:10% PS:5%PIP_2	Martini3 (CG)	10 µs x 2	unbiased
SMP Y257A	80% PC:20% PE	Martini3 (CG)	10 µs x 4	unbiased
SMP F258A	80% PC:20% PE	Martini3 (CG)	10 µs x 4	unbiased
SMP F258A	85% PC:10% PS:5%PIP_2	Martini3 (CG)	10 µs x 4	unbiased
SMP R256E	80% PC:20% PE	Martini3 (CG)	10 µs x 4	unbiased
SMP R256E	85% PC:10% PS:5%PIP_2	Martini3 (CG)	10 µs x 4	unbiased
Control	none	Martini3 (CG)	10 µs x 2	unbiased
SMP	none	CHARMM36m (AA)	1 µs	unbiased
SMP	none	CHARMM36m (AA)	50 ns x 2	biased
SMP	80% PC:20% PE	CHARMM36m (AA)	1 ns	equilibrium
