# Supplementary material for: Insights into membrane association of the SMP domain of extended synaptotagmin
Source: Nat Commun. 2023 Mar 17;14:1504. doi: 10.1038/s41467-023-37202-8 (PMC10023780; doi:10.1038/s41467-023-37202-8)
Supplement: Supplementary file 2 — Description of Additional Supplementary Files [file 41467_2023_37202_MOESM2_ESM.pdf]

**Description of Additional Supplementary Files**

File Name: Supplementary Movie 1
Description: The movie was obtained from an adiabatic bias molecular dynamics (ABMD) simulation in which a ratchet-andpawl-like potential was added on the center of mass of the headgroup of the bound POPE lipid to accelerate its motion from one tip to the other. It showed that the loaded lipid could slide along the hydrophobic groove of one SMP, but couldn’t pass through the SMP dimer interface and even dropped off from the binding pocket of SMP, indicating a huge energy barrier at the interface of the SMP dimer.
