# Supplementary material for: Insights into membrane association of the SMP domain of extended synaptotagmin
Source: Nat Commun. 2023 Mar 17;14:1504. doi: 10.1038/s41467-023-37202-8 (PMC10023780; doi:10.1038/s41467-023-37202-8)
Supplement: Supplementary file 4 — Reporting Summary [file 41467_2023_37202_MOESM4_ESM.pdf]

nature portfolio

Corresponding author(s): Xin Bian; Yong Wang

Last updated by author(s): Feb 13, 2022

Reporting Summary

Nature Portfolio wishes to improve the reproducibility of the work that we publish. This form provides structure for consistency and transparency in reporting. For further information on Nature Portfolio policies, see our Editorial Policies and the Editorial Policy Checklist.

Statistics

For all statistical analyses, confirm that the following items are present in the figure legend, table legend, main text, or Methods section.

n/a	Confirmed	
☐	☒	The exact sample size (*n*) for each experimental group/condition, given as a discrete number and unit of measurement
☐	☒	A statement on whether measurements were taken from distinct samples or whether the same sample was measured repeatedly
☐	☒	The statistical test(s) used AND whether they are one- or two-sided<br>*Only common tests should be described solely by name; describe more complex techniques in the Methods section.*
☒	☐	A description of all covariates tested
☒	☐	A description of any assumptions or corrections, such as tests of normality and adjustment for multiple comparisons
☐	☒	A full description of the statistical parameters including central tendency (e.g. means) or other basic estimates (e.g. regression coefficient) AND variation (e.g. standard deviation) or associated estimates of uncertainty (e.g. confidence intervals)
☐	☒	For null hypothesis testing, the test statistic (e.g. *F*, *t*, *r*) with confidence intervals, effect sizes, degrees of freedom and *P* value noted<br>*Give P values as exact values whenever suitable.*
☒	☐	For Bayesian analysis, information on the choice of priors and Markov chain Monte Carlo settings
☒	☐	For hierarchical and complex designs, identification of the appropriate level for tests and full reporting of outcomes
☒	☐	Estimates of effect sizes (e.g. Cohen's *d*, Pearson's *r*), indicating how they were calculated

Our web collection on statistics for biologists contains articles on many of the points above.

Software and code

Policy information about availability of computer code

Data collection	AMT Image Capture Engine, SoftMax Pro 5.2, Typhoon FLA 9500, ImagJ 1.51s., Gromacs 2021.5, PLUMED 2.7.4
Data analysis	ImagJ 1.51s, GraphPad Prism 8.0., VMD 1.9.4, PyMOL 2.5.0, CHARMM-GUI server

For manuscripts utilizing custom algorithms or software that are central to the research but not yet described in published literature, software must be made available to editors and reviewers. We strongly encourage code deposition in a community repository (e.g. GitHub). See the Nature Portfolio guidelines for submitting code & software for further information.

Data

Policy information about availability of data

All manuscripts must include a data availability statement. This statement should provide the following information, where applicable:

- Accession codes, unique identifiers, or web links for publicly available datasets
- A description of any restrictions on data availability
- For clinical datasets or third party data, please ensure that the statement adheres to our policy

All raw data are available from the corresponding authors upon reasonable request. The source data underlying Figures 1b, 1d, 1g, 2b-d, 3a-b, 3d-e, 4b-c, 5g-m, 6b and Supplementary Figures 1b-d, 2d-e, 3a, 4, 5a, 7, 8, 10a are provided as a Source Data file. The crystal structure of SMP domain of human E-Syt2 has previously been deposited in PDB: 4P42 [http://doi.org/10.2210/pdb4P42/pdb].

Human research participants

Policy information about studies involving human research participants and Sex and Gender in Research.

Reporting on sex and gender	N/A
Population characteristics	N/A
Recruitment	N/A
Ethics oversight	N/A

Note that full information on the approval of the study protocol must also be provided in the manuscript.

Field-specific reporting

Please select the one below that is the best fit for your research. If you are not sure, read the appropriate sections before making your selection.

☒ Life sciences ☐ Behavioural & social sciences ☐ Ecological, evolutionary & environmental sciences

For a reference copy of the document with all sections, see nature.com/documents/nr-reporting-summary-flat.pdf

Life sciences study design

All studies must disclose on these points even when the disclosure is negative.

Sample size	Sample size was not predetermined by any statistical method. Sample sizes per group were estimated based on the previously published papers (Yang et al, 2021, PMID: 33785892; Nascimbeni, 2017, PMID: 28550152).
Data exclusions	No data were excluded from the analyses.
Replication	All experiments were repeated at least 3 times with similar results.
Randomization	No experiments that require randomization are performed in this study.
Blinding	No experiments that require blinding are performed in this study.

Reporting for specific materials, systems and methods

We require information from authors about some types of materials, experimental systems and methods used in many studies. Here, indicate whether each material, system or method listed is relevant to your study. If you are not sure if a list item applies to your research, read the appropriate section before selecting a response.

Materials & experimental systems

n/a	Involved in the study	
☒	☐	Antibodies
☐	☒	Eukaryotic cell lines
☒	☐	Palaeontology and archaeology
☒	☐	Animals and other organisms
☒	☐	Clinical data
☒	☐	Dual use research of concern

Methods

n/a	Involved in the study	
☒	☐	ChIP-seq
☒	☐	Flow cytometry
☒	☐	MRI-based neuroimaging

Eukaryotic cell lines

Policy information about cell lines and Sex and Gender in Research

Cell line source(s)	Expi293 cells (purchased from Thermo Fisher Scientific A14527), Hela cells (purchased from ATCC CCL-2).
Authentication	Beyond purchase from reputable source, no further validation was carry out.
Mycoplasma contamination	We routinely do the mycoplasma contamination test and the cell lines were tested negative.
Commonly misidentified lines (See ICLAC register)	No commonly misidentified line was used.
